# The Effects of Mechanical Preload on Transmural Differences in Mechano-Calcium-Electric Feedback in Single Cardiomyocytes: Experiments and Mathematical Models

**DOI:** 10.3389/fphys.2020.00171

**Published:** 2020-03-17

**Authors:** Anastasia Khokhlova, Pavel Konovalov, Gentaro Iribe, Olga Solovyova, Leonid Katsnelson

**Affiliations:** ^1^Institute of Immunology and Physiology, Russian Academy of Sciences, Yekaterinburg, Russia; ^2^Institute of Natural Sciences and Mathematics, Ural Federal University, Yekaterinburg, Russia; ^3^Department of Physiology, Asahikawa Medical University, Hokkaido, Japan; ^4^Department of Cardiovascular Physiology, Okayama University, Okayama, Japan

**Keywords:** single cardiomyocytes, mechanical preload, length-dependent activation, transmural differences, electromechanical coupling, mechano-calcium-electric feedback

## Abstract

Transmural differences in ventricular myocardium are maintained by electromechanical coupling and mechano-calcium/mechano-electric feedback. In the present study, we experimentally investigated the influence of preload on the force characteristics of subendocardial (Endo) and subepicardial (Epi) single ventricular cardiomyocytes stretched by up to 20% from slack sarcomere length (SL) and analyzed the results with the help of mathematical modeling. Mathematical models of Endo and Epi cells, which accounted for regional heterogeneity in ionic currents, Ca^2+^ handling, and myofilament contractile mechanisms, showed that a greater slope of the active tension–length relationship observed experimentally in Endo cardiomyocytes could be explained by greater length-dependent Ca^2+^ activation in Endo cells compared with Epi ones. The models also predicted that greater length dependence of Ca^2+^ activation in Endo cells compared to Epi ones underlies, via mechano-calcium-electric feedback, the reduction in the transmural gradient in action potential duration (APD) at a higher preload. However, the models were unable to reproduce the experimental data on a decrease of the transmural gradient in the time to peak contraction between Endo and Epi cells at longer end-diastolic SL. We hypothesize that preload-dependent changes in viscosity should be involved alongside the Frank–Starling effects to regulate the transmural gradient in length-dependent changes in the time course of contraction of Endo and Epi cardiomyocytes. Our experimental data and their analysis based on mathematical modeling give reason to believe that mechano-calcium-electric feedback plays a critical role in the modulation of electrophysiological and contractile properties of myocytes across the ventricular wall.

## Introduction

Over the past 30 years, the main concept of the studies on regional heterogeneity in the healthy heart has maintained that transmural differences are required in the myocyte function to effectively optimize the ejection of blood by the heart (Katz and Katz, 1989; Solovyova et al., 2016). Transmural differences in action potential (AP) properties have been reported at the single-cell level in humans (Johnson et al., 2018), and large (Cordeiro et al., 2004) and small (Chen et al., 2017) animals. Endocardial and subendocardial (Endo) cardiomyocytes across species have a higher AP upstroke velocity, longer AP duration (APD) and smaller phase-1 repolarization rate as compared with epicardial and subepicardial (Epi) myocytes. A larger Na^+^ current, *I*_*Na*_, in the subendocardium underlies the higher AP upstroke velocity in Endo cells, whereas a greater transient outward current, *I*_*to*_, and a smaller L-type Ca^2+^ current, *I*_*CaL*_, and Na^+^–Ca^2+^ exchanger current, *I*_*N*__*aCa*_, both (Wan et al., 2005; Xu et al., 2012) underlie the higher repolarization rate and shorter APD in Epi cells (Veerman et al., 2017).

These differences in repolarization duration lead to Endo-Epi differences in the Ca^2+^ transient and force developed. On the other hand, regional heterogeneity in the expression and function of Ca^2+^ cycling and sarcomere proteins, including L-type Ca^2+^ channels (Wang and Cohen, 2003; Wan et al., 2005), SERCA2a (Lou et al., 2011), Na^+^–Ca^2+^ exchanger (Dilly et al., 2006), and myosin isoform (Cazorla et al., 2005), also contribute to regional variations in the Ca^2+^ transient and sarcomere shortening. Experiments on single cells showed that Endo myocytes had a higher diastolic Ca^2+^ level, greater amplitude of the Ca^2+^ transient and sarcomere shortening, and slower rising and decline phases of the Ca^2+^ transient and sarcomere shortening (Cordeiro et al., 2004; Pan et al., 2018). Moreover, single studies on mechanically loaded cardiomyocytes demonstrated that the Frank–Starling mechanisms depended on cell location with a steeper sarcomere length (SL)–active tension relationship (Cazorla et al., 1997, 2000) and a larger increase in Ca^2+^ sensitivity following stretch (Cazorla et al., 2005) in endocardium than in epicardium. They also revealed a positive gradient of cellular stiffness in intact cardiomyocytes across the ventricular wall from Epi to Endo. Our previous results suggested that heterogeneity in the parameters of Ca^2+^ handling and myofilament mechanics between Endo and Epi cardiomyocytes via cooperative mechanisms of mechano-calcium-electric feedback modulates transmural differences in AP properties between the cells (Khokhlova et al., 2018a). Thus, there are several cross-links, electromechanical, mechano-calcium, and mechano-electric, that underlie the manifestation of transmural heterogeneity in the cellular function.

In the intact heart, the transmural gradient in both diastolic stretch and systolic contraction was revealed in the canine ventricle, with the greatest stretch and contraction in the Endo layer (Waldman et al., 1985). Experiments *in vivo* showed that myofiber shortening in the intact canine ventricle started earliest at the Endo layer and was progressively delayed toward the Epi one, whereas the onset of myofiber relaxation occurred earlier in epicardium than endocardium (Ashikaga et al., 2007).

Although the authors discovered a regional dispersion of myofiber shortening in the intact heart, they found no evidence of significant transmural gradient in electrical repolarization (Ashikaga et al., 2007). It was suggested that the transmural activation pattern compensates for the intrinsic transmural difference in APD, resulting in more homogeneous repolarization of the intact ventricular wall (Opthof et al., 2007; Myles et al., 2010; Boukens et al., 2015). Moreover, some studies showed no transmural gradients in APD in intact hearts (Taggart et al., 2001; Mačianskienė et al., 2018). Thus, the link between the intracellular mechanisms of transmural heterogeneity at the cellular level and its role in the whole heart function are poorly understood.

Previously, we showed that mechanical preload affects the transmural gradient in cell contractile properties, and even a small diastolic stretch (∼3.5% from slack SL) reduces transmural differences in the time to peak contraction and decay time of the Ca^2+^ transient between Endo and Epi cardiomyocytes (Khokhlova et al., 2018b). In the present study, we investigate the force characteristics of Endo and Epi single ventricular cardiomyocytes from the mouse heart using an original single-cell stretch technique (Iribe et al., 2014) to stretch cells by up to 20% from slack SL. To test the hypothesis that mechanical preload may influence the transmural gradient in the electrophysiological function of Endo and Epi cells, we present here detailed mathematical models of Endo and Epi cardiomyocytes, which account for regional heterogeneity in ionic currents, Ca^2+^ handling, and myofilament contractile mechanisms.

## Materials and Methods

### Isolation of Mouse Ventricular Myocytes

All procedures involving animal use were performed in accordance with the Guiding Principles for the Care and Use of Animals approved by the Council of the Physiological Society of Japan. All protocols involving animals were approved by the Animal Subjects Committee of Okayama University, Graduate School of Medicine, Dentistry and Pharmaceutical Sciences.

Endo and Epi myocytes were isolated from healthy adult (8- to 10-week-old) male C57BL6/J mice by enzymatic dissociation and mechanical dispersion as described previously (Khokhlova et al., 2018b). Briefly, hearts were excised from mice sacrificed by an overdose of isoflurane (DS Pharma Animal Health, Osaka, Japan), mounted on a Langendorff perfusion apparatus, and perfused retrogradely through the aorta with oxygenated Ca^2+^-free solution supplemented with 10 mM 2,3-butanedione monoxime (Sigma-Aldrich, St. Louis, MO, United States) for 4–6 min, followed by the same solution containing 0.093 mg/ml Liberase TM (Roche, Basel, Switzerland) and 12.5 μM CaCl_2_ for 6–7 min at 37°C. Following the perfusion, the central region of left ventricular (LV) free wall was separated in the latter solution using fine forceps and scissors and then tissue pieces from the Endo and Epi layers of the LV free wall were dissected under a microscope. All separation procedures were carried out at room temperature (22–24°C). Then, the Endo and Epi tissue pieces were separately minced and incubated at 37°C for 5 min in fresh enzyme buffer, subsequently dispersed by gentle trituration and placed into Ca^2+^-free solution supplemented with 12.5 μM CaCl_2_ and 10% fetal bovine serum (FBS; Sigma-Aldrich, United States). The resulting cell suspensions were filtered and centrifuged once for 3 min at 15 × *g* (Kubota, Tokyo, Japan). Then, cardiomyocytes were consistently resuspended in Ca^2+^-containing solution (0.6, 1.2, and 1.8 mM) and harvested by gravity sedimentation. Finally, single myocytes were stored in Hepes-buffered Tyrode solution containing (in mM): NaCl (140), KCl (5.4), CaCl_2_ (1.8), MgCl_2_ (1.0), Hepes (5), and glucose (11) (pH adjusted to 7.4 using NaOH) at room temperature until used within 8 h of cell isolation. The cardiomyocytes selected for study showed clear sarcomere patterns and were quiescent when not stimulated.

### Measurements of Single Cell Length and Force

The detailed method to provide length and force measurements on single cardiomyocytes is described elsewhere (Iribe et al., 2014; Khokhlova et al., 2018b). In brief, two pairs of carbon fibers (CF) (∼10 μm in diameter, Tsukuba Materials Information Laboratory, Ltd., Tsukuba, Japan) were attached to the top and bottom surfaces of the left and right cell end, while both CFs on the left side were mounted on computer-controlled piezoelectric transducers (PZT). To change the mechanical preload (to apply end-diastolic axial stretch to a cell), both left CFs received the same movement command (in our protocol, 2 μm per 300 ms for a step) using LabVIEW (National Instruments Corp., Austin, TX, United States), while both right CFs rigidly fixed the right cell end, preventing it from moving. The distance between left and right CF tips (“cell length”) and SL changes were recorded during uncontrolled auxotonic contraction (when a cell contracted under mechanical loading by CFs) using the IonOptix equipment and software (IonOptix Corporation, Milton, MA, United States). All experiments were performed at a stimulation frequency of 1 Hz at room temperature. After all CFs were set, a cell was paced at 1 Hz for 3 min before recordings to enhance cell–CF adhesion (Iribe et al., 2007, 2014).

Cellular force was calculated as follows: *F* = *K* ⋅ (Δ*L*_*PZT*_ −Δ*L*_*CF*_), where *K* is the combined stiffness of the left CFs (0.07–0.09 μN/μm in this study), Δ*L*_*PZT*_ is the change in PZT position, and Δ*L*_*CF*_ is the change in distance between left and right CF tips. Then, force was normalized to effective cross-sectional area, assuming an elliptical shape of the cross section with a 3:1 ratio of long (measured cell width, y-direction) and short axes (estimated cell height, *z*-direction) (Nishimura et al., 2004).

The SL range across the ventricular wall and its changes during the cardiac cycle are still unknown. Chung and Granzier reported that end-diastolic SL (EDSL) varied in a transmural-dependent manner in the contracted isolated mouse heart (2.08 ± 0.01 μm in epicardium and 1.98 ± 0.02 μm in endocardium) (Chung and Granzier, 2011). Under physiological settings at high heartbeat frequencies in mice *in vivo*, the EDSL value in the epicardial surface was 1.97 ± 0.20 μm (Kobirumaki-Shimozawa et al., 2016). In this study, we stretched single cardiomyocytes by up to 20% from slack SL, i.e., up to ∼2.16 μm to cover the physiological range of EDSL in cardiomyocytes found in the above studies.

### Analysis of Length-Dependent Changes in Cellular Force and Time Characteristics

To analyze length-dependent changes in cellular force, we measured the slopes of the end-diastolic force–length relationship curve (EDFLR), end-systolic force–length relationship curve (ESFLR), and active force–length relationship curve (*F*_*active*_LR). The amplitudes of passive (end-diastolic), total (end-systolic), and active (total minus passive) tension were fitted by linear regression (*R*^2^ > 0.9) for relative changes in cell length (see Supplementary Material and Supplementary Figure S1). We also used an index called “Frank–Starling Gain” (FSG), calculated as the ratio of ESFLR and EDFLR slopes (Bollensdorff et al., 2011). Being a dimensionless and cross-section independent parameter, it is not sensitive to errors in force normalization and supports improved inter-individual comparison of cells.

**FIGURE 1:**
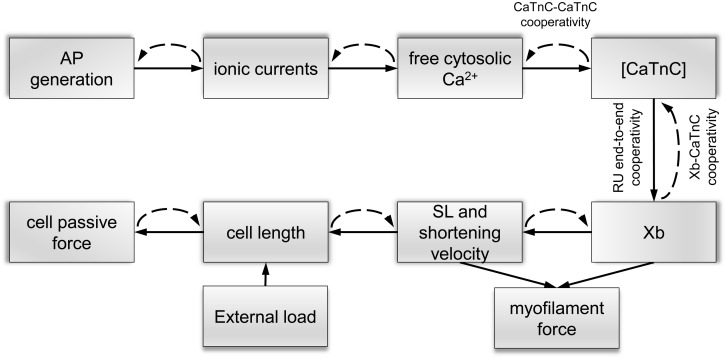
Schematic links between mechanisms of electromechanical coupling and mechano-calcium-electric feedback in the mathematical Endo and Epi models. Solid lines show direct links between the mechanisms of excitation–contraction coupling and dashed lines show feedback links. Cooperative mechanisms of Ca^2+^ activation of myofilaments [Xb–CaTnC, CaTnC–CaTnC, and regulatory unit (RU) end-to-end cooperativity] are described in the text.

Time to peak contraction (*T*_max_) was measured from stimulation onset to the peak of auxotonic tension. Time to 50% relaxation (TR_50_) was measured as the time interval from *T*_max_ to the time of 50% relaxation. To analyze the effects of preload on the time characteristics of contraction and relaxation, we assessed the dependence of *T*_max_ and TR_50_ on the relative changes in the end-diastolic cell length. We found that only *T*_max_LR (*T*_max_–length relation) curve could be fitted well by linear regression (*R*^2^ > 0.8). No correlation was found between TR_50_ and changes in the end-diastolic cell length.

### Statistical Analysis

Data analysis was carried out with Origin 8.0 (Origin Lab, Northampton, MA, United States) and GraphPrism 7.0 (GraphPad Software, San Diego, CA, United States). The data are expressed as the mean ± standard error of the mean (SEM). A Student’s unpaired *t* test was used for statistical analysis. Differences between means with *p* < 0.05 were considered statistically significant.

### Mathematical Modeling

Previously, we developed mathematical models of Endo and Epi cells based on the Yekaterinburg–Oxford mathematical model (Sulman et al., 2008). These models reproduce transmural differences in the contractile function of mouse single ventricular cardiomyocytes at room temperature (Khokhlova et al., 2018b). To mimic Endo–Epi differences in the electrophysiological parameters and mechano-calcium-electric feedback, we have incorporated our contraction model into the models of electrical activity (Pandit et al., 2001) and Ca^2+^ handling (Hinch et al., 2004). After analysis of several available models for the electrophysiological characteristics of rodent cardiomyocytes, the models that we had adopted gave us the best fit to literature and our experimental data. The Pandit model was initially developed for rat Endo and Epi cardiomyocytes. We have therefore adjusted the model parameters (Table 1) to fit literature data on AP generation and Ca^2+^ transient and our experimental data on Endo/Epi myocyte contraction obtained for mice at room temperature.

**TABLE 1:** Literature and experimental data on the Endo/Epi differences taken into account in the mathematical models.

**Target**	**Endo**	**Epi**	**Animal**	**Source**
Na^+^ current (*I*_*Na*_) amplitude	Bigger	Smaller	Mouse	Veerman et al., 2017
Transient outward K^+^ current (*I*_*t*_) amplitude	Smaller	Bigger	Mouse	Rossow et al., 2006; Liu et al., 2015; Veerman et al., 2017
Na^+^–Ca^2+^ exchanger current (*I*_*Na*__*Ca*_) amplitude	Smaller	Bigger	Mouse	Dilly et al., 2006; Xu et al., 2012
Ca^2+^ release flux from sarcoplasmic reticulum (*J*_*RyR)*_ amplitude	Bigger	Smaller	Mouse	Dilly et al., 2006
SERCA2a Ca^2^ pump flux (*J*_*SERCA*_) amplitude	Bigger	Smaller	Mouse	Pan et al., 2018
Rate constant of Xb cycling	Smaller	Bigger	Guinea pig Pig	Le Guennec et al., 2008 Stelzer et al., 2008
Passive stiffness	Bigger	Smaller	Mouse	Experimental data
Probability *n*_1_(*l*_1_) of Xb attachment to the actin filament	Bigger	Smaller	Mouse	Experimental data

A key feature of our single-cell models is that it includes mechano-calcium-electric cross-links. The cooperative mechanisms of Ca^2+^ activation of myofilament regulatory units (RUs) were formulated and justified in our previous papers (Katsnelson et al., 2004; Khokhlova et al., 2018b). Here, we describe them briefly (Figure 1):

#### Xb–CaTnC Cooperativity

The rate of CaTnC dissociation decreases with an increase in the fraction of force generating cross-bridges (Xbs) per single CaTnC complex.

#### CaTnC–CaTnC Cooperativity

The rate of CaTnC complex dissociation decreases with increasing numbers of CaTnC complexes.

#### RU End-to-End Cooperativity

Ca^2+^ binding by TnC, located within one RU on a thin filament affects the neighboring RUs through tropomyosin end-to-end conformational interaction, thus contributing to the opening of the active actin sites for myosin head attachment.

The non-linear dependence of the on-rate for Xb attachment on the concentration of CaTnC complexes [CaTnC] (via RU end-to-end cooperativity, see Supplementary Material 2.5.9, M_*A*_) and the off-rate for CaTnC dissociation on the fraction of Xbs (via Xb–CaTnC cooperative mechanism, see Supplementary Material 2.4.12, π_*NA*_) and [CaTnC] (via CaTnC–CaTnC cooperative mechanism, see Supplementary Material 2.4.13, e^kA*CaTnC^) formalize the key cooperative mechanisms of myofilament Ca^2+^ activation in the models (Figure 1). These three mechanisms underlie a number of essential properties of the cardiac muscle, such as the dependence of contraction and relaxation on myofilament length and load (Katsnelson et al., 2004).

In our cell models, the key link that ties the mechano-calcium feedback together with the cooperativity mechanisms is the mechanosensitive probability *n*_1_(*l*_1_) of Xb attachment to the actin filament depending on the instantaneous SL: the bigger *l*_1_ (the deformation of the sarcomere against its slack length), the higher *n*_1_ (see Supplementary Material 2.5.9, *n*_1_). In short, *n*_1_(*l*_1_) accounts for the effect of length on Xb fraction, while Xb–CaTnC cooperativity accounts for the effect of Xb fraction on [CaTnC]. The other two types of cooperativity amplify this effect. Note that our phenomenological dependence *n*_1_(*l*_1_) is invariant with respect to the underlying intracellular mechanisms, which are still debated (see section “Discussion”).

The rheological scheme of the models consists of a cardiomyocyte attached to an extra-series element (see Supplementary Material and Supplementary Figure S2, XSE), which corresponds to CFs in experiments. The elastance of the extra-series element corresponds to CF stiffness in experimental settings. The sum of the cell and extra-series element lengths is set to be constant, while the cell length dynamically changes during the cardiac cycle under the loading of the extra-series elastic element, i.e., under the auxotonic condition.

Euler integration with a time step of 0.01 ms and the Newton method for solving algebraic equations were used for simulations. All the results are shown in their steady state, achieved by allowing the models to run for 30 s at a stimulation frequency of 1 Hz.

To compare model simulation with experimental data, we fitted passive, total, and active tension amplitudes as well as the *T*_max_, amplitude of the Ca^2+^ transient, time from peak to 50% and 90% Ca^2+^ decay (CaT_50_, CaT_90_), and APD at 90% repolarization (APD_90_) at varying preload by linear regression lines for relative changes in cell length.

## Results

### The Transmural Gradient in AP, Ca^2+^ Transient, Force Development and the Cell Morphology

We found no differences in the morphological parameters between unstretched single Endo and Epi cells. Cell length (Endo: 120.0 ± 2.0 μm, *n* = 56 vs. Epi: 120.7 ± 1.8 μm, *n* = 57), width (22.75 ± 0.33 μm, *n* = 56 vs. 22.21 ± 0.28 μm, *n* = 57), and slack EDSL (Endo: 1.78 ± 0.01 μm, *n* = 34 vs. Epi: 1.77 ± 0.01 μm, *n* = 28) were similar between the groups.

**FIGURE 2:**
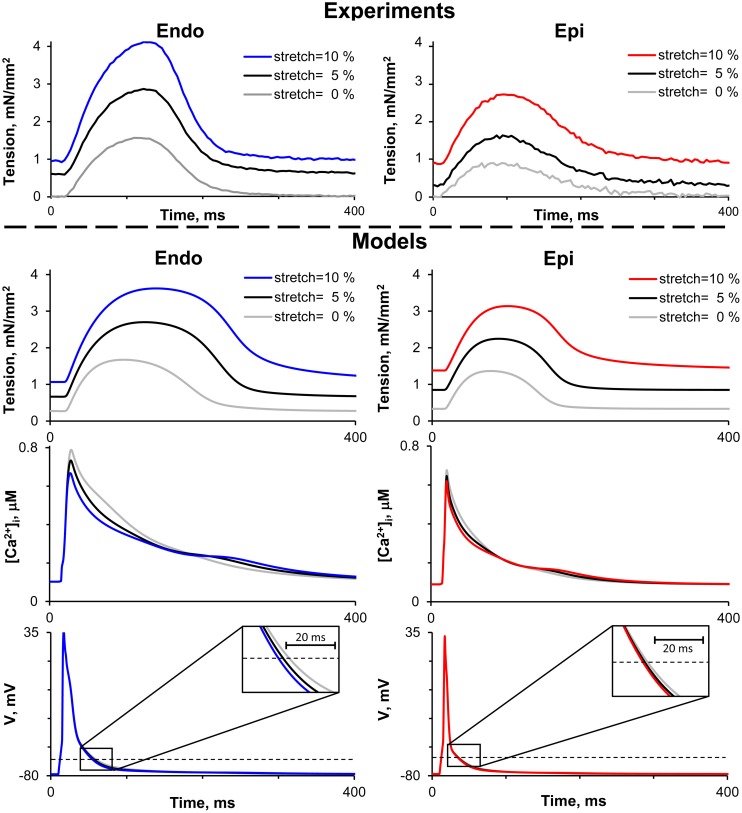
Representative recordings of auxotonic tension in experiments (top panel) and mathematical model simulations of tension, cytosolic Ca^2+^ concentration ([Ca^2+^]_*i*_) and action potential (V) during the cardiac cycle (bottom panel) of Endo and Epi isolated cardiomyocytes at varying preload (cell stretch 5 and 10% relative to the initial end-diastolic length).

**FIGURE 3:**
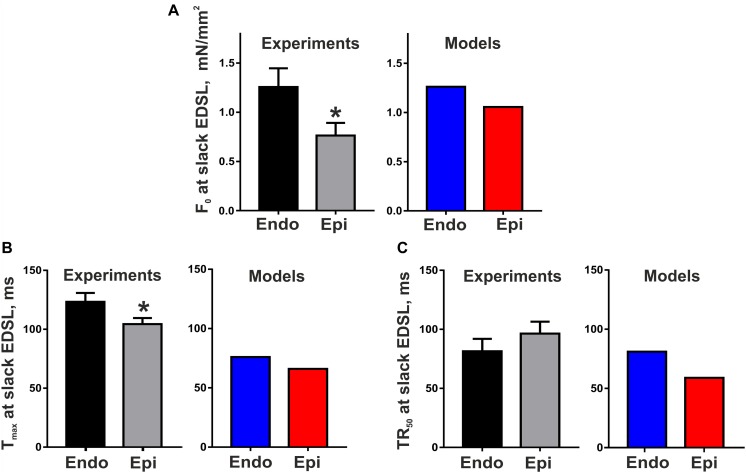
Transmural gradient in the parameters of mouse cardiomyocyte contraction at slack end-diastolic sarcomere length (EDSL) in experiments [Endo: *n* = 8 (*N* = 3), Epi: *n* = 9 (*N* = 5)] and mathematical models. **(A)** Tension amplitude (*F*_0_), **(B)** Time to peak contraction (*T*_max_), **(C)** Time to 50% relaxation (TR_50_). *p < 0.05 Endo vs. Epi, Student’s unpaired *t* test. *N* = number of animals, *n* = number of cells.

Figure 2 shows representative recordings of auxotonic tension in the experiments and mathematical model simulations at varying preload. Comparing the parameters of cell contractions at slack EDSL, we found the amplitude of auxotonic tension to be higher and the time to peak contraction (*T*_max_) to be greater in Endo cells compared with Epi ones (Figures 2, 3). No significant difference was found in the time to 50% relaxation (TR_50_) between the cells (Figure 3).

The Endo and Epi mathematical models described above were able to reproduce the experimental data on transmural differences in contraction parameters (Figure 3). A comparison of the AP and Ca^2+^ transient characteristics in the models with literature data obtained for unloaded mouse single cardiomyocytes showed good consistency (Table 2). Resting membrane potential was similar in Endo and Epi cells. APD was greater in Endo than in Epi cardiomyocytes. Endo cardiomyocytes also showed a slower Ca^2+^ transient. Consistently with literature data (Dilly et al., 2006; Rossow et al., 2006; Pan et al., 2018), the Endo model had a higher Ca^2+^ transient amplitude (15% relative to the Endo model) and a higher diastolic Ca^2+^ level (12% relative to the Epi model) compared with the Epi model (Table 2).

**TABLE 2:** Experimental and simulated parameters of action potential, Ca^2+^ transient, and contraction for Endo and Epi mouse ventricular myocytes.

	**Experiment, RT 1 Hz**	**Simulation, RT 1 Hz**	**Shi et al. (2013), RT 1 Hz**	**Brunet et al. (2004), RT 1 Hz**	**Khokhlova et al. (2018b), RT 1 Hz**	**Kondo et al. (2006), RT 1 Hz**	**Dilly et al. (2006), 37°C 1 Hz**	**Pan et al. (2018), 37°C 1 Hz**	**Chen et al. (2017), 37°C 8 Hz**
**Endo**									
RP (mV)		−78.80 (0.2%)		−70 ± 2 (0%)					
APD_90_ (ms)		52 (40%)	36.0 ± 2.0 (58%)	18.0 ± 2.8 (12%)			93.1 ± 5.4 (53%)		56.2 ± 6.4 (9%)
Diastolic [Ca^2+^]_*i*_ (μM or *F*_340_/*F*_380_ ratio)		0.101 μM (12%)					0.256 ± 0.020 μM (42%)	0.52 ± 0.023 ratio (13%)	
Ca amplitud (μM or *F*_340_/*F*_380_ ratio)		0.705 μM (15%)			0.105 ± 0.017 ratio (24%)		0.630 ± 0.088 μM (43%)	0.241 ± 0.014 ratio (16%)	
CaTR_50_ (ms)		65 (57%)			137.0 ± 4.4 (8%)		194.3 ± 8.2 (6%)		
CaTR_90_ (ms)		215 (38%)						469.6 ± 22.4 (18%)	
Tension amplitud (mN/mm^2^)	1.26 ± 0.19 (39%)	1.12 (18%)							
*T*_max_ (ms)	123 ± 7 (15%)	76 (13%)						58.4 ± 3.7 (18%)	
TR_50_ (ms)	81 ± 10 (-18%)	81 (19%)				98 ± 3 (-5%)			
TR_90_ (ms)		138 (34%)						215.5 ± 39.8 (46%)	
**Epi**									
RP (mV)		−78.6		−70 ± 2					
APD_90_ (ms)		31	15.1 ± 1.2	15.8 ± 2.5			43.8 ± 6.1		51.2 ± 5.8
Diastolic [Ca^2+^]_*i*_ (μM or *F*_340_/*F*_380_ ratio)		0.089 μM					0.148 ± 0.18 μM	0.45 ± 0.022 ratio	
Ca amplitud (μM or *F*_340_/*F*_380_ ratio)		0.599 μM			0.080 ± 0.012 ratio		0.358 ± 0.91 μM	0.203 ± 0.011 ratio	
CaTR_50_ (ms)		28			126.0 ± 4.9		183.1 ± 4.8		
CaTR_90_ (ms)		133						384.4 ± 19.7	
Tension amplitud (mN/mm^2^)	0.77 ± 0.11	0.919							
*T*_max_ (ms)	104 ± 5	66						47.6 ± 2.2	
TR_50_ (ms)	96 ± 10	59				103 ± 3			
TR_90_ (ms)		91						115.8 ± 18.5	

*RP, resting potential; APD_90_, action potential duration at 90% repolarization; [Ca^2+^]_*i*_, cytosolic Ca^2+^ concentration; CaT_50_ and CaT_90_, time from peak to 50% and 90% Ca^2+^ decay; *T*_*max*_, time to peak contraction; TR_50_ and TR_90_, time to 50% and 90% relaxation. The relative percentage of differences between the Endo and Epi models relative to the Endo model is shown in parentheses.*

### The Effects of Preload on the Transmural Gradient in Length-Dependent Activation and Time Course of Contraction

**FIGURE 4:**
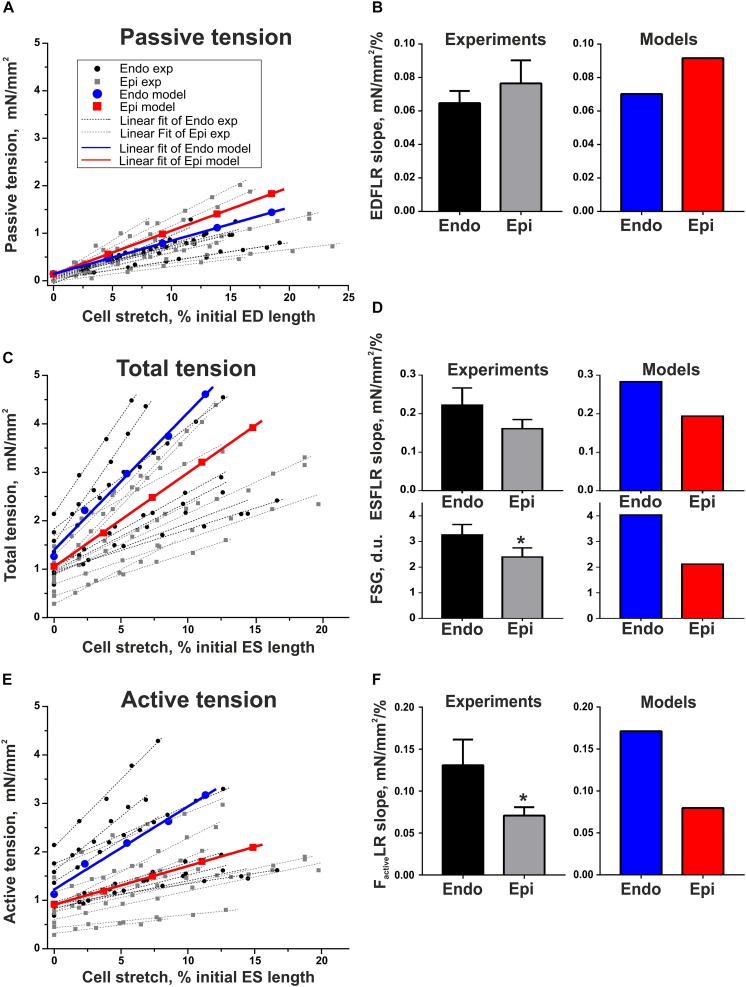
Length-dependent changes in passive, total, and active (total minus passive) tension of single mouse ventricular Endo and Epi cardiomyocytes in experiments [Endo: *n* = 8 (*N* = 3), Epi: *n* = 9 (*N* = 5)] and mathematical models. **(A)** End-diastolic force–length relation curves (EDFLR) from experimental (exp) data set and model simulations. **(B)** EDFLR slopes as the stiffness index in experiments and mathematical models. **(C)** End-systolic force–length relation curves (ESFLR) from experimental (exp) data set and model simulations. **(D)** ESFLR slopes and FSG index (the ratio of ESFLR and EDFLR slopes) in experiments and mathematical models. **(E)** Active force–length relation curves (*F*_*active*_LR) from experimental (exp) data set and model simulations. **(F)** *F*_*active*_LR slopes in experiments and mathematical models. *p* < 0.05 Endo vs. Epi, Student’s unpaired *t* test. *N* = number of animals, *n* = number of cells. ED, end-diastolic; ES, end-systolic.

To estimate length-dependent activation in Endo and Epi cardiomyocytes, we analyzed the slopes of the end-diastolic force–length relationship curve (EDFLR), end-systolic force–length relationship curve (ESFLR), active force–length relationship curve (*F*_*active*_LR), and FSG index expressed as the ratio of ESFLR and EDFLR slopes (see Supplementary Material and Supplementary Figure S1). We found no significant difference in the slope of EDFLR between Endo and Epi cells, demonstrating their similar passive stiffness (Figure 4). The parameters of the mathematical models for passive force description were fitted to mimic experimental values (Supplementary Table S2.2 in Supplementary Material, Figure 4).

Neither did we find any significant difference in the ESFLR slope between the groups; however, the FSG index and *F*_*active*_LR slope were significantly higher in Endo cells than in Epi cells, pointing to greater myofilament length-dependent activation in Endo cardiomyocytes (Figure 4). By setting the mechanosensitive probability *n*_1_(*l*_1_) of Xb attachment to the actin filament in the Endo model greater than in the Epi one, we were able to reproduce the experimental data (Supplementary Table S2.2 in Supplementary Material and Figure 4).

**FIGURE 5:**
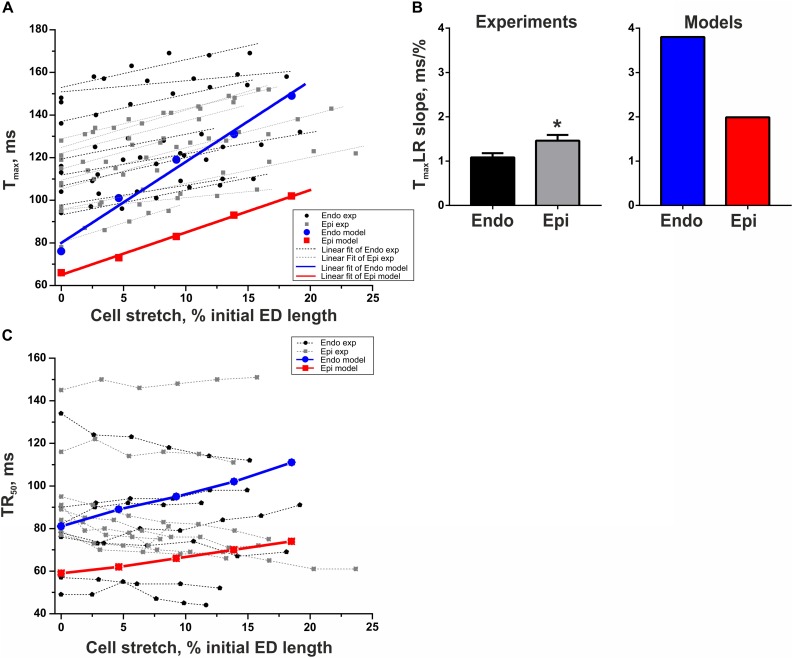
Length-dependent changes in the time to peak contraction (*T*_max_) and time to 50% relaxation (TR_50_) of single mouse ventricular Endo and Epi cardiomyocytes in experiments [Endo: *n* = 8 (*N* = 3), Epi: *n* = 9 (*N* = 5)] and mathematical models. **(A)** *T*_max_–length relation curves (*T*_max_LR) from experimental (exp) data set and model simulations. **(B)** *T*_max_LR slopes in experiments and mathematical models. **(C)** No correlation was found between TR_50_ and changes in the end-diastolic cell length in experiments. *p < 0.05 Endo vs. Epi, Student’s unpaired *t* test. *N* = number of animals, *n* = number of cells. ED, end-diastolic.

Analyzing the effects of preload on the time course of contraction, we found that *T*_max_ increased with preload in Epi cells significantly greater that in Endo ones, resulting in a decrease in the transmural gradient in *T*_max_ at high preload (Figure 5). Previously, we had suggested that a greater deceleration of CaTnC dissociation at increased preload in Epi cells may contribute to a steeper preload dependence of *T*_max_ in these cells (Khokhlova et al., 2018b). In the present study, our mathematical models, accounting for new experimental data on transmural differences in length-dependent activation (*F*_*active*_LR slope and FSG index), failed to reproduce the steeper *T*_max_–length relationship in Epi cells (Figure 5). Possible explanations will be discussed below. In contrast to *T*_max_, the experiments did not reveal any significant change in TR_50_ with an increase in preload in either Endo or Epi cells; however, both the Endo and Epi mathematical models predicted a monotonic rise in TR_50_.

It is also interesting that in the analysis of the tension–length relationship, the two populations of cells from the same Endo or Epi region were visually distinguishable (Figures 4, 5).

### The Effects of Preload on the Transmural Gradient in Excitation–Contraction Coupling

**FIGURE 6:**
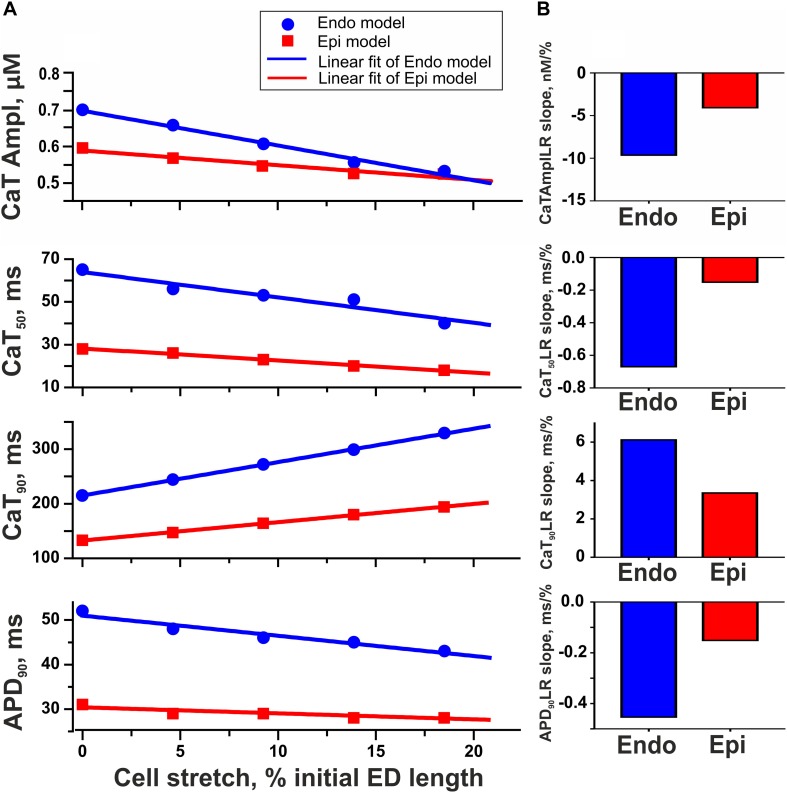
Model predictions of length-dependent changes in the amplitude of Ca^2+^ transient (CaT Ampl), time from peak to 50% and 90% Ca^2+^ decay (CaT_50_, CaT_90_), and action potential duration at 90% repolarization (APD_90_) in Endo and Epi cells. **(A)** CaAmplLR, CaT_50_LR, Ca_90_LR, and APD_90_LR—Ca^2+^ transient amplitude–length relation, CaT_50_–length relation, CaT_90_–length relation, and APD_90_–relation curves, respectively. **(B)** The slopes of CaAmplLR, CaT_50_LR, CaT_90_LR, and APD_90_LR. ED, end-diastolic.

To test the hypothesis that mechanical preload affects the transmural gradient in excitation–contraction coupling between Endo and Epi cells, we estimated the parameters of Ca^2+^ transient and AP in the mathematical models of Endo and Epi cardiomyocytes. An increase in preload (20% diastolic stretch) decreased the amplitude of the Ca^2+^ transient in both the Endo and Epi models, the decrease being more pronounced for Endo cells (24% in the Endo model vs. 12% in the Epi model, Figure 6). No effect of stretch on the AP amplitude was found in both cell-type models. Regarding the time course parameters, stretch had almost no effect on the time to peak of the Ca^2+^ transient in both models, but a greater decrease in CaT_50_ with a greater increase in Ca_90_ was found in the Endo model compared with the Epi one at high preload (Figure 6). These steeper length-dependent changes with an increase in preload in the cytosolic Ca^2+^ decay were accompanied by a greater decrease in APD_90_ in the Endo model in comparison with the Epi one (17% in the Endo model vs. 10% in the Epi model), providing a reduction of the transmural gradient in APD_90_ at high preload between Endo and Epi cardiomyocytes (Figure 6). Analysis of the models showed that a decrease in the Ca^2+^ transient amplitude and acceleration of the fast phase of the cytosolic Ca^2+^ decay with an increase in preload resulted in Na^+^–Ca^2+^ exchanger activity modulation. The outward Na^+^–Ca^2+^ exchange current increased (0.7% in Endo vs. 0.1% in Epi for peak amplitudes), while the inward Na^+^–Ca^2+^ exchange current decreased (21% in Endo vs. 17% in Epi for peak amplitudes). These changes led to an acceleration of repolarization, which, in turn, slightly increased voltage-dependent outward K^+^ currents (∼2% in Endo vs. ∼0.5% in Epi for peak amplitudes) and accelerated its activation. Thus, greater changes in cytosolic Ca^2+^ with an increase in preload in the Endo model resulted in greater modulation of the Na^+^–Ca^2+^ exchange current and voltage-dependent outward K^+^ currents compared with the Epi model causing greater shortening of APD in Endo cardiomyocytes.

## Discussion

This study was designed to examine the effects of mechanical preload on mechano-calcium-electric feedback in single cardiomyocytes isolated from the Endo and Epi layers of the central region of mouse free LV wall. Using an original single-cell stretch technique (Iribe et al., 2014), we stretched single cardiomyocytes by up to 20% from slack SL to confirm the results of our previous study suggesting steeper length-dependent changes in *T*_max_ in Epi cells compared with Endo cells under small preloads (Khokhlova et al., 2018b). For the purposes of the study, we developed detailed mathematical models of electromechanical coupling in mouse Endo and Epi cardiomyocytes, which reproduced our experimental data on smaller active tension–length relationship (*F*_*active*_LR) in Epi cardiomyocytes compared with Endo ones. The models showed that this effect could be explained by greater length-dependent Ca^2+^ activation in Endo cells. However, the Epi model failed to reproduce the bigger slope of the *T*_max_–length relationship simultaneously with the smaller slope of *F*_*active*_LR compared with the Endo model. We speculate that preload-dependent changes in viscosity should be involved alongside the Frank–Starling effects to regulate the transmural gradient in length-dependent changes in the time course of contraction between Endo and Epi cardiomyocytes.

Using our Endo and Epi mathematical models, we studied the effects of preload on the transmural gradient in APD between the Endo and Epi cardiomyocytes. The models predicted that greater length-dependent activation in Endo cells *via* mechano-calcium-electric feedback gives a greater decrease in APD with stretch compared with Epi cells, providing a reduction in the transmural gradient in APD at longer end-diastolic SL.

### The Transmural Gradient in Myocyte Morphology and Contractile Function

Our findings of similar length, width, and EDSL for mouse LV Endo and Epi cardiomyocytes are consistent with data obtained for rats (Cazorla et al., 2000; Natali et al., 2002; McCrossan et al., 2004).

Consistent with findings on mouse unloaded single-cell shortening (Kondo et al., 2006; Pan et al., 2018), our data showed that the amplitude of auxotonic tension was higher and *T*_max_ was greater in single Endo cells compared with Epi ones. The Endo and Epi mathematical models that we developed for the study reproduced the experimental data on transmural differences in contraction and literature data on the transmural gradient in AP and Ca^2+^ parameters (Table 2).

The discrepancies in the time of cytosolic Ca^2+^ decay between the models and experiments could also be explained by the binding characteristics of the fluorescent dyes used for Ca^2+^ measurements. These dyes may have contributed to cytosolic Ca^2+^ kinetics that is not accounted for in the mathematical models.

We also found that the time of tension decay was longer in the Endo model (negligibly for TR_50_) but shorter in the Epi model compared with the experimental data. These discrepancies could be explained in two ways. First, the models overlooked the additional mechanism of intracellular transmural heterogeneity, which mostly affected the relaxation phase. The second explanation can be associated with the substantial variation of relaxation time with a standard deviation of 28% of the mean for Endo cells and 26% for Epi cells despite the fact that we used only the central part of the LV avoiding edge effects from the base and apex. Interestingly, in earlier studies, the variation of contractile properties among cardiomyocytes within small regions was also found to be significant (Clark and Campbell, 2019; Clark et al., 2019). Thus, within a rat Epi myocardial tissue volume of 7 mm^3^, RT_50_ was found to have a standard deviation of 28% of the mean, and this degree of variation persisted even within tissue volumes as small as 1 mm^3^ (Clark and Campbell, 2019). The authors discovered that RT_50_ was directly related to TnI phosphorylation levels and correlated significantly with end-diastolic cell length, suggesting a link between cell morphology and intrinsic relaxation behavior.

In our study, we observed two populations of cardiomyocytes with regard to their force–length properties between cells from the same region, i.e., within the endocardium and within the epicardium. Previously, Hanft and McDonald found that cells isolated from the rat LV exhibited two populations of force–length relationships (Hanft and McDonald, 2010). Thus, our findings are in agreement with the observation that functional variability among individual myocytes at the microscale may contribute to the contractile properties of the myocardium (Clark and Campbell, 2019; Clark et al., 2019). However, in the intact heart, the electrotonic coupling of cardiomyocytes via gap junctions and mechanical connections between cells at costameres and intercalated discs may attenuate these cell-to-cell differences at the macroscale (McCain and Parker, 2011; Quinn et al., 2016).

In this study, we used a parametrically modified Pandit model, which initially described the electrophysiological activity of Endo and Epi rat cardiomyocytes (Pandit et al., 2001), and a simplified model of Ca^2+^ handling in ventricular myocytes (Hinch et al., 2004). The choice of the models was determined by good coupling with our contractile model of Endo and Epi mouse cardiomyocytes (Khokhlova et al., 2018b) and best fit to literature and experimental data. To our knowledge, there are models (Bondarenko and Rasmusson, 2010; Mullins and Bondarenko, 2013) that account for transmural differences in electromechanical coupling in mice. In contrast to those models, our study takes into account not only literature data on transmural differences in intracellular ionic mechanisms and Ca^2+^ cycling but also intrinsic differences in acto-myosin interaction that may underlie Endo/Epi differences in mice (Table 1). We failed to find direct data on regional differences in murine Xb cycling kinetics and so had to use data obtained for guinea pigs and pigs (Le Guennec et al., 2008; Stelzer et al., 2008), which together with other target mechanisms allowed us to reproduce the functional electromechanical behavior of mouse Endo and Epi cardiomyocytes.

### The Effects of Preload on the Transmural Gradient in Mechano-Calcium-Electric Feedback

To analyze changes in force amplitude and time course of contraction in a wide range of preloads, we used a modified CF technique where two pairs of CFs are used like two forceps for fixing and stretching a cell along the long axis (Iribe et al., 2014).

To analyze length-dependent changes in the force of Endo and Epi cardiomyocytes, we measured the slopes of the EDFLR as an index of myocardial passive stiffness and the slopes of the ESFLR, *F*_*active*_LR, and FSG index as indices of a cell’s ability to generate force under mechanical load. Consistently with our previous results (Khokhlova et al., 2018b), we found that there was no significant difference in the slope of the EDFLR and ESFLR between Endo and Epi cells. However, an assessment of the active tension–length relationship and the FSG index showed that the slope of *F*_*active*_LR and the FSG index were significantly higher in Endo cardiomyocytes than in Epi ones, indicating greater myofilament length-dependent activation in Endo cells. Observations of rat cardiomyocytes also revealed a steeper active tension–SL relationship in Endo cells against Epi ones (Cazorla et al., 2000). Experiments on rat skinned cells demonstrated a larger increase in myofibrillar Ca^2+^ sensitivity following stretch due to a higher phosphorylation level of myosin-light chain 2 after stretch in Endo cells than in Epi cells (Cazorla et al., 2005, 2006).

There are still debates going concerning the mechanisms underlying increased contractility upon preload/stretch (length-dependent activation). It has been suggested that a decrease in myofilament lattice spacing between thin and thick filaments (McDonald and Moss, 1995; Irving et al., 2000; Fukuda et al., 2003) due to an increase in SL results in an increased number of attached Xbs, which, in turn, cooperatively increases the affinity of troponin C for Ca^2+^ (Hofmann and Fuchs, 1987) enhancing thin filament activation. Recent studies have shown that other mechanisms may be involved in this order of events instead of myofilament lattice spacing, e.g., changes in myosin head orientation relative to thin filaments (Farman et al., 2011) and titin-based structural rearrangements within both thin and thick filaments (Ait-Mou et al., 2016). In our models, the phenomenologically described length-dependent probability *n*_1_(*l*_1_) of Xb binding to actin (see Supplementary Material, 2.5.9, *n*_1_) is invariant with respect to a particular mechanism providing an increase in *n*_1_ in response to an increase in *l*_1_; this mechanism may be involved in addition to or instead of lattice spacing. The setting of *n*_1_(*l*_1_) in the Endo model greater than in the Epi one enabled us to reproduce the experimental data on transmural differences in the slope of F_*active*_LR and the FSG index.

In our mathematical models, an increase in preload increases the Xb formation probability *n*_1_(*l*_1_). An increase in the concentration of force-generating Xbs brings about an increase in TnC affinity for Ca^2+^ (the first type of cooperativity implemented through a decrease in the rate constant of Ca^2+^ dissociation from Ca^2+^–TnC complexes). Furthermore, the higher the concentration of [CaTnC], the lower the off-rate (the second type of cooperativity), which leads to a subsequent slowing down of the Ca^2+^–TnC decay. As a consequence, Ca^2+^–TnC binding has a higher peak and decreases slowly at longer SL (Solovyova et al., 2003). The slowing down of Ca^2+^–TnC dissociation leads to a slight decrease in the amplitude and acceleration of the fast phase of the cytosolic Ca^2+^ decay (Figure 6, CaT_50_).

By stretching single cardiomyocytes by up to 20% from slack SL, we have now confirmed the finding obtained in our previous study for small stretch (Khokhlova et al., 2018b): Epi cardiomyocytes demonstrate steeper length-dependent changes in *T*_max_ compared with Endo cardiomyocytes. Previously, we suggested that a greater slowing down of CaTnC dissociation at increased preload in Epi cells may contribute to a steeper preload dependence of *T*_max_ in Epi ones (Khokhlova et al., 2018b). In this study, while accounting for new experimental data on transmural differences in *F*_*active*_LR and FSG index, our mathematical models failed to reproduce the bigger slope of the *T*_max_–length relationship simultaneously with the smaller slope of *F*_*active*_LR in Epi cells. As model analysis showed, a greater deceleration of CaTnC dissociation in Epi cells that could give a steeper *T*_max_–length relationship with stretching would lead to a bigger slope of *F*_*active*_LR in these cells. The latter would directly contradict the experimentally obtained steeper F_*active*_LR in Endo cells. Thus, the models suggest that the deceleration of CaTnC dissociation at increased preload should be greater in Endo cells rather than in Epi ones. This means that other mechanisms should be involved in the regulation of the transmural gradient in length-dependent changes in the time course of contraction of Endo and Epi cardiomyocytes. For example, stretch-hold experiments involving mice and pigs showed that viscosity based on interaction between titin and actin depends on stretch velocity and EDSL, being greater at longer SL (Chung and Granzier, 2011; Chung et al., 2011a, b). In our previous modeling study, an increase in viscosity decreased *T*_max_ of contraction but did not affect length-dependent activation (Katsnelson et al., 2004). It has been shown on rats that the amount of titin differed between the subendocardium and subepicardium, with Endo cells expressing more titin per half-sarcomere than Epi cells (Cazorla et al., 2005). Our preliminary simulations have confirmed that a greater viscosity response to an increase in preload in the Endo model accounts for a smaller increase in *T*_max_ at longer SL, i.e., for a smaller slope of the *T*_max_–length relationship in Endo cardiomyocytes. At the same time, *F*_*active*_LR remains unaffected by a change in viscosity, i.e., remains greater in Endo cardiomyocytes due to the greater slowing down of CaTnC dissociation at high preload in Endo cells. These preliminary findings should be verified in future studies.

It is still unclear how an increase in preload affects the transmural gradient in AP characteristics. The only study available on isolated rat Endo and Epi cardiomyocytes showed that transmural electrophysiological responses to stretch are different (Saint et al., 2010). The authors suggested that a greater expression of TREK-1, a mechanosensitive K^+^ channel, may be accountable for a greater decrease in the APD in Endo cells compared with Epi ones upon stretch, providing a decrease of the transmural gradient in APD. Our models predict one more possible mechanism of this phenomenon mediated via mechano-calcium-electric feedback.

In the Endo model, a greater Xb formation probability *n*_1_(*l*_1_) provides a greater increase in TnC affinity for Ca^2+^ compared with the Epi model and a greater acceleration of CaT_50_, respectively (Figure 6, CaT_50_). This, in turn, leads to a greater modulation of the Na^+^–Ca^2+^ exchange current and outward K^+^ currents causing a greater shortening of APD in the Endo model at longer SL (Figure 6, APD). Thus, we suggest that Endo/Epi heterogeneity in length-dependent activation via mechano-calcium-electric feedback provides a reduction in the transmural gradient in APD at longer SL, which may partially explain the difficulties of determination of transmural gradients in APD in the intact heart (Taggart et al., 2001; Mačianskienė et al., 2018).

In conclusion, transmural heterogeneity is a major characteristic of the healthy LV. We have demonstrated that a change in preload might be a source for mechano-calcium-electric modulation of electrophysiological and contractile properties of myocytes across the wall.

### Mathematical Model Limitation

In the mathematical models, we did not aim to precisely mimic the mean values of experiments or literature data. Our goal was to ensure falling into the correct parameter range to be able to explain the molecular and cellular mechanisms that underlie transmural heterogeneity in the heart. However, some output parameters, such as *T*_max_, turned out to be out of the range. The tuning mathematical model parameters should be continued in further studies.

## Data Availability Statement

The datasets generated for this study are available on request to the corresponding author.

## Ethics Statement

All procedures involving animal use were performed in accordance with the Guiding Principles for the Care and Use of Animals approved by the Council of the Physiological Society of Japan. All protocols involving animals were approved by the Animal Subjects Committee of Okayama University, Graduate School of Medicine, Dentistry and Pharmaceutical Sciences.

## Author Contributions

AK and GI contributed to the design of wet experiments, analysis, and interpretation of the results. PK contributed to the mathematical model simulations and analysis. OS and LK contributed to the conception of the mathematical models, design, analysis, and interpretation of the results and simulations. The manuscript was written by AK, with the assistance of GI, OS, and LK. All authors approved the final version of the manuscript.

## Conflict of Interest

The authors declare that the research was conducted in the absence of any commercial or financial relationships that could be construed as a potential conflict of interest.
