# Supplementary material for: The Effects of Mechanical Preload on Transmural Differences in Mechano-Calcium-Electric Feedback in Single Cardiomyocytes: Experiments and Mathematical Models
Source: Front Physiol. 2020 Mar 17;11:171. doi: 10.3389/fphys.2020.00171 (PMC7091561; doi:10.3389/fphys.2020.00171)
Supplement: Supplementary file 1 [file Data_Sheet_1.PDF]

Supplementary Material

1 ANALYSIS OF LENGTH-DEPENDENT CHANGES IN CELLULAR FORCE IN EXPERIMENTS

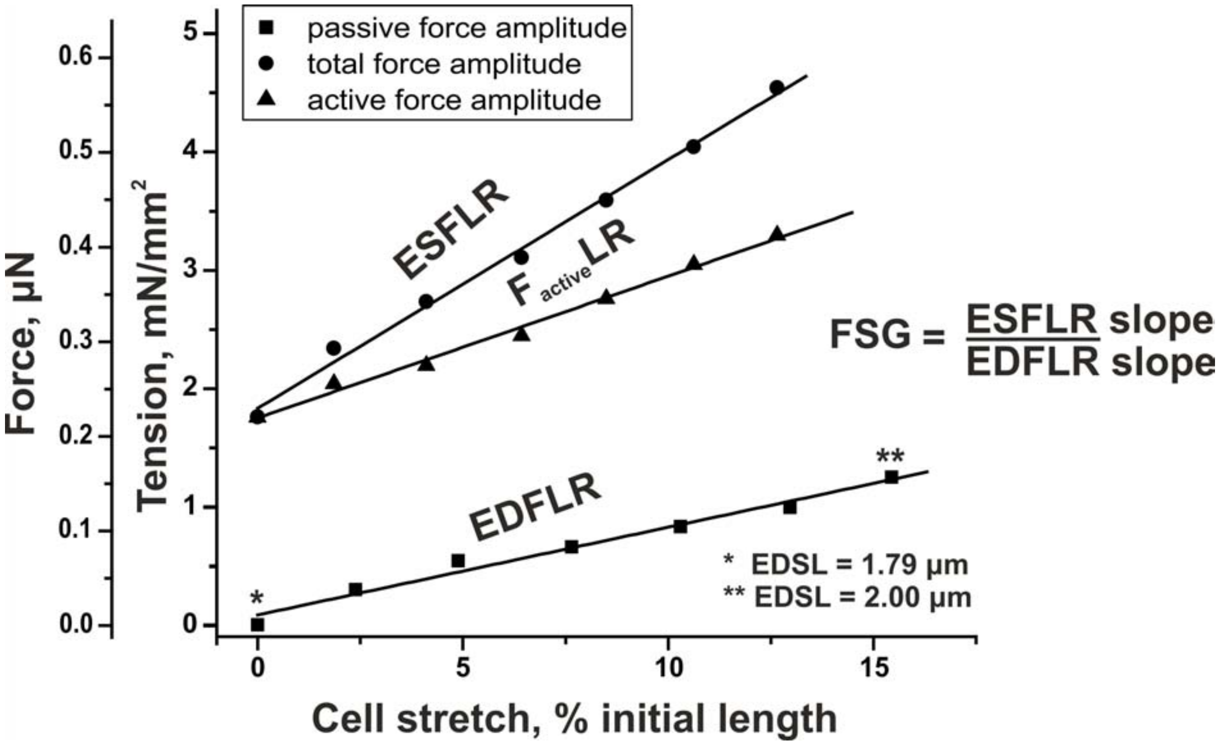

Figure S1. Representative recording of the force-length dependence of a single cardiomyocyte contracting at varying preload. The end-diastolic force-length relation (EDFLR), end-systolic force-length relation (ESFLR), and active force-length relation ($F_{active}LR$) are fitted by linear regression lines of amplitudes of auxotonic passive (end-diastolic), total (end-systolic), and active (total minus passive) tension for relative changes in the cell length expressed as a percentage of the initial cell length (end-diastolic for EDFLR, end-systolic for ESFLR and $F_{active}LR$, respectively). "Frank–Starling Gain" (FSG) is calculated as the ratio of ESFLR and EDFLR slopes.

2 PANDIT-HINCH+EKATERINBURG (PH+E) MODEL DESCRIPTION

The single Endo and Epi cardiomyocyte model equations and formulations based on the Pandit (Pandit et al. (2001)), Hinch (Hinch et al. (2004)) and Ekaterinburg-Oxford mathematical model (EO model, Sulman et al. (2008)) are presented here. Pandit and Hinch modified equations are taken from Terkildsen, Niederer, Cramper and Smith model (Terkildsen et al. (2008)).

2.1 Variables

2.1.1 State variables

	Definition	Init.Value Endo	Init.Value Epi	Unit
V	Membrane potential	-78.8	-78.62	mV
K_i	Intracellular K^+ concentration	138.479	139.196	mM
Na_i	Intracellular Na^+ concentration	11.41	10.58	mM
Ca_i	Intracellular Ca^{2+} concentration	$1.014 \cdot 10^{-4}$	$8.9367 \cdot 10^{-5}$	mM
Ca_{SR}	SR Ca^{2+} concentration	1.005580	1.693392	mM
$CaTnC$	Ca^{2+}-troponin C complexes concentration	0.003285	0.002888	mM
z_1	Combined state of the CaRU	0.98821	0.98809	dimensionless
z_2	Combined state of the CaRU	0.00874	0.00862	dimensionless
z_3	Combined state of the CaRU	0.003019	0.003245	dimensionless
r	Activation gating variable (i_t)	0.002540	0.002582	dimensionless
s	Fast inactivation gating variable (i_t)	0.974985	0.983802	dimensionless
s_{slow}	Slow inactivation gating variable (i_t)	0.568106	0.736642	dimensionless
y	Inactivation gating variable (i_f)	0.002992	0.003097	dimensionless
h	Fast inactivation gating variable (i_{Na})	0.609702	0.602331	dimensionless
j	Slow inactivation gating variable (i_{Na})	0.609289	0.602201	dimensionless
m	Activation gating variable (i_{Na})	0.005479	0.005639	dimensionless
r_{ss}	Activation gating variable (i_{ss})	0.003353	0.003407	dimensionless
s_{ss}	Inactivation gating variable (i_{ss})	0.279792	0.285481	dimensionless
N	Cross-bridges concentration	$2.6 \cdot 10^{-6}$	$4.1 \cdot 10^{-7}$	dimensionless

2.1.2 Mechanical variables

	Definition	Init.Value Endo	Init.Value Epi	Unit
l_1	Deformation of CE against its slack length (for $EDSL = 1.84\mu m$)	0.13024	0.12936	μm
l_2	Deformation of PE against its slack length (for $EDSL = 1.84\mu m$)	0.13029	0.1294	μm
l_3	Deformation of XSE against its slack length (for $EDSL = 1.84\mu m$)	0.04	0.046	μm
v	velocity of CE deformation	0	0	μm/ms
w	velocity of PE deformation	0	0	μm/ms

2.2 Constants

	Definition	Value Endo	Value Epi	Unit Unit
Cm	Total membrane capacitance	10^{-4}		μ F
F	Faraday constant	96487		C/mole
R	Universal gas constant	8314.5		mJ/mole · K
T	Temperature	295		kelvin
$stim_{amplitude}$	amplitude of i_{stim}	$-6 \cdot 10^{-4}$		μA
$stim_{duration}$	duration of i_{stim}	10		ms
$stim_{period}$	periodicity of i_{stim}	1000		ms
V_{SR}	Volume of sarcoplasmic reticulum	$2.098 \cdot 10^{-6}$		μL
V_{myo}	Myoplasmic volume	25850		μm^3
Ca_o	Extracellular Ca^{2+} concentration	1.2		mM
K_o	Extracellular K^+ concentration	5.4		mM
Na_o	Extracellular Na^+ concentration	140		mM
g_{CaB}	Maximum conductance (I_{CaB})	$2.6875 \cdot 10^{-8}$		mM/mV · ms
K_{mCa}	Ca^{2+} Half saturation (NCX)	$1.19 \cdot 10^{-3}$		mM
K_{mNa}	Na^+ Half saturation (NCX)	88.8		mM
eta	Voltage dependence of NCX control	0.35		dimensionless
g_{NCX}	Pump rate (NCX)	$4.15 \cdot 10^{-2}$		mM/ms
k_{sat}	Low potential saturation factor (NCX)	0.1		dimensionless
K_{mpCa}	Half saturation of sarcolemmal Ca^{2+} pump	$5 \cdot 10^{-4}$		mM
g_{pCa}	Maximum pump rate of sarcolemmal pump	$3.5 \cdot 10^{-6}$		mM/ms
B_{CMDN}	Total cytosolic calmodulin concentration	0.05		mM
k_{CMDN}	Half saturation constant of calmodulin	$2.382 \cdot 10^{-3}$		mM
a_{iKt}	Parameter of I_t current	0.7345	0.886	dimensionless
b_{iKt}	Parameter of I_t current	0.2655	0.114	dimensionless
g_t	Maximum conductance (I_t)	$3.2 \cdot 10^{-5}$	$5 \cdot 10^{-5}$	mS
g_{B_K}	Maximum conductance (I_{B_K})	$1.38 \cdot 10^{-7}$		mS
$g_{B_{Na}}$	Maximum conductance ($I_{B_{Na}}$)	$8.015 \cdot 10^{-8}$		mS
f_{Na}	Fraction of Na^+ channels (i_f)	0.2		dimensionless
g_f	Maximum conductance (i_f)	$1.45 \cdot 10^{-6}$		mS
g_{K1}	Maximum conductance (i_{K1})	$2.4 \cdot 10^{-5}$		mS
g_{Na}	Maximum conductance ($i_N a$)	$8.15 \cdot 10^{-4}$	$6.15 \cdot 10^{-4}$	mS
K_{m_K}	Half-maximum K^+ binding constant (i_{NaK})	1.5		mM
$K_{m_{Na}}$	Half-maximum Na^+ binding constant (i_{NaK})	10		mM
$i_{NaK_{max}}$	Maximum i_{NaK} current	$9.5 \cdot 10^{-5}$		μA
g_{ss}	Maximum conductance (I_{ss})	$7 \cdot 10^{-6}$		mS
K_L	Half Concentration of inactive LCC (CaRU)	$0.22 \cdot 10^{-3}$		mM

CONSTANTS (CONTINUED)

	Definition	Value Endo	Value Epi	Unit
K_{RyR}	Half concentration of RyR channel activation (CaRU)	$41 \cdot 10^{-3}$		mM
V_L	Potential when half LCC open (CaRU)	-2		mV
a	Biasing to make inactivation function of V (CaRU)	0.0625		dimensionless
b	Biasing to make inactivation function of V (CaRU)	14		dimensionless
c	Biasing to make inactivation function of $[Ca^{2+}]_{ds}$ (CaRU)	0.01		dimensionless
d	Biasing to make inactivation function of $[Ca^{2+}]_{ds}$ (CaRU)	100		dimensionless
δ_{VL}	Width of opening potentials (CaRU)	7		mV
ϕ_L	Proportion of time closed in open mode (CaRU)	2.35		dimensionless
ϕ_R	Proportion of time closed in open mode (CaRU)	0.05		dimensionless
t_L	Time switching between C and O states (CaRU)	1		ms
τ_L	Inactivation time (CaRU)	650		ms
τ_R	Inactivation time (CaRU)	2.43		ms
θ_R	Reciprocal of proportion of time inactivated in open mode (CaRU)	0.012		dimensionless
J_L	Permeability of single LCC (CaRU)	$6.15 \cdot 10^{-4}$		μm^3/ms
J_R	Permeability of single RyR (CaRU)	0.02		μm^3/ms
N_{RU}	Number of release units (CaRU)	65000	62000	dimensionless
g_D	Ca^{2+} flux rate from dyadic space to cytosol	0.065		μm^3/ms
K_{SERCA}	Half saturation of SERCA	$5 \cdot 10^{-4}$		mM
g_{SERCA}	Maximum pump rate of SERCA	$8 \cdot 10^{-3}$	$1.5 \cdot 10^{-3}$	mM/ms
g_{SRl}	Rate of leak from the SR to cytosol	$1.8951 \cdot 10^{-5}$		ms^{-1}
Π_{min}	Parameter of Π_{N_A} function	0.03		dimensionless
f_A	Parameter of $N_A(CaTnC, N)$ function	0.8		dimensionless
TnC_{tot}	Total concentration of TnC	0.07		mM
k_A	Cooperativity parameter	20.0		mM^{-1}
a_{off}	Maximum rate constant for $CaTnC$ dissociation	44.0		ms^{-1}
a_{on}	Rate constant for $CaTnC$ association	20.0		mM$^{-1}\cdot$ ms^{-1}
λ	Scale parameter of F_{CE}	60		mN/mm^2
α_1	Exponential coefficient of F_{SE}	25		μm^{-1}
β_1	Linear coefficient of F_{SE}	2.14664	0.4713	mN/mm^2
α_2	Coefficient of F_{PE}	3.9721	5.198	μm^{-1}

CONSTANTS (CONTINUED)

	Definition	Value Endo	Value Epi	Unit
β_2	Linear coefficient of F_{PE}	-0.2476	-0.3409	mN/mm^2
α_3	Coefficient of F_{XSE}	17.8475	22.8451	μm^{-1}
β_3	Linear coefficient of F_{XSE}	-0.4538	-0.7320	mN/mm^2
α_{vp_l}	Exponential coefficient of F_{VS_1}	16.0		μm^{-1}
α_{vp_s}	Exponential coefficient of F_{VS_1}	29.0		μm^{-1}
β_{vp_l}	Linear coefficient of F_{VS_1}	$1.67 \cdot 10^{-4}$		mN/$mm^2$$\cdot$ ms/μm
β_{vp_s}	Linear coefficient of F_{VS_1}	$1.67 \cdot 10^{-1}$		mN/$mm^2$$\cdot$ ms/μm
α_{vs_l}	Exponential coefficient of F_{VS_2}	46.0		μm^{-1}
α_{vs_s}	Exponential coefficient of F_{VS_2}	46.0		μm^{-1}
β_{vs_l}	Linear coefficient of F_{VS_2}	0		mN/$mm^2$$\cdot$ ms/μm
β_{vs_s}	Linear coefficient of F_{VS_2}	0		mN/$mm^2$$\cdot$ ms/μm
v_{max}	Parameter of p function	0.005		μm/ms
a_p	Parameter of p function	0.25		dimensionless
d_h	Parameter of P_{star} function	0.5		dimensionless
x_1	Parameter of P_{star} function	0.07857		dimensionless
α_P	parameter of G_{star} function	4.0		dimensionless
α_G	parameter of G_{star} function	1.0		dimensionless
k_μ	parameter of M_A function	0.6		dimensionless
μ	parameter of M_A function	3		dimensionless
g_1	parameter of n_1 function	2.35	1.8	μm^{-1}
g_2	parameter of n_1 function	0.03	0.403	dimensionless
$n1_A$	parameter of n_1 function	1.8	0.85	dimensionless
$n1_B$	parameter of n_1 function	32	28	μm
$n1_C$	parameter of n_1 function	1	1	dimensionless
$n1_Q$	parameter of n_1 function	0.9	0.9	dimensionless
$n1_K$	parameter of n_1 function	1.135	1.135	dimensionless
$n1_\nu$	parameter of n_1 function	0.04	0.04	dimensionless
S_0	parameter of L_{oz} function	1.14		μm
S_{055}	parameter of L_{oz} function	0.55		μm
S_{046}	parameter of L_{oz} function	0.46		μm
κ	parameter of κ function	0.625	0.69	dimensionless
κ_0	parameter of κ function	2.8	4	dimensionless
m_0	fraction of strongly attached Xb	0.9		dimensionless
q_1	parameter of q function	0.0173		ms^{-1}
q_2	parameter of q function	0.259		ms^{-1}
q_3	parameter of q function	0.0173		ms^{-1}
q_4	parameter of q function	0.02		ms^{-1}
α_Q	parameter of q function	10.0		dimensionless
β_Q	parameter of q function	5.0		dimensionless
x_{st}	parameter of q function	0.7576		dimensionless

CONSTANTS (CONTINUED)

	Definition	Value Endo	Value Epi	Unit
$r0$	preload (for $EDSL = 1.84\mu m$)	0.27038	0.33185	mN/mm^2

*: CaRU - Ca^{2+} Release unit (Hinch model)

MODEL EQUATIONS

2.3 Pandit, Hinch block (with modifications) of the model

2.3.1 Membrane potential

$$\frac{\mathrm{d}V}{\mathrm{d}time} = \frac{-\left(I_{Na} + I_t + I_{ss} + I_f + I_{K1} + I_{B_{Na}} + I_{B_K} + I_{NaK} + I_{Stim} + I_{CaB} + I_{NaCa} + I_{pCa} + I_{LCC}\right)}{Cm}$$

$$i_{Stim} = \begin{cases} -stim_{amp} & \text{if } \left(time - \lfloor \frac{time}{stim_{per}} \rfloor \cdot stim_{per} \geq stim_{start}\right) \text{ and} \\ & \text{and } \left(time - \lfloor \frac{time}{stim_{per}} \rfloor \cdot stim_{per} \leq stim_{start} + stim_{dur}\right) \\ 0 & \text{otherwise} \end{cases}$$

2.3.2 Intracellular ion concentrations

$$\frac{\mathrm{d}Na_i}{\mathrm{d}time} = \frac{-\left(I_{Na} + I_{B_{Na}} + I_{NaCa} \cdot 3 + I_{NaK} \cdot 3 + I_{f_{Na}}\right) \cdot 1}{V_{myo_{uL}} \cdot F}$$

$$\frac{\mathrm{d}K_i}{\mathrm{d}time} = \frac{-\left(I_{Stim} + I_{ss} + I_{B_K} + I_t + I_{K1} + I_{f_K} + -2 \cdot I_{NaK}\right) \cdot 1}{V_{myo_{uL}} \cdot F}$$

$$\frac{\mathrm{d}Ca_i}{\mathrm{d}time} = \beta_{CMDN} \cdot \left(I_{RyR} - I_{SERCA} + I_{SR} - \frac{\mathrm{d}CaTnC}{\mathrm{d}time} - \frac{-2 \cdot I_{NaCa} + I_{pCa} + I_{CaB} + I_{LCC}}{2 \cdot V_{myo_{uL}} \cdot F}\right)$$

$$\frac{\mathrm{d}Ca_{SR}}{\mathrm{d}time} = \frac{V_{myo_{uL}}}{V_{SR_{uL}}} \cdot \left(-I_{RyR} + I_{SERCA} - I_{SR}\right)$$

2.3.3 Variable convertion from Hinch model to Pandit model

$$I_{LCC} = -1.5 \cdot I_{LCC}(Hinch) \cdot 2 \cdot V_{myo_{uL}} \cdot F$$

$$I_{NaCa} = I_{NaCa}(Hinch) \cdot V_{myo_{uL}} \cdot F$$

$$I_{pCa} = I_{pCa}(Hinch) \cdot 2 \cdot V_{myo_{uL}} \cdot F$$

$$I_{CaB} = -I_{CaB}(Hinch) \cdot 2 \cdot V_{myo_{uL}} \cdot F$$

$$I_{RyR} = 1.5 \cdot I_{RyR}(Hinch)$$

2.3.4 Background Ca^{2+} current

$$E_{Ca} = \frac{R \cdot T}{2 \cdot F} \cdot \ln \frac{Ca_o}{Ca_i}$$

$$I_{CaB}(Hinch) = g_{CaB} \cdot (E_{Ca} - V)$$

2.3.5 Na^{+}-Ca^{2+} Exchanger

$$I_{NaCa}(Hinch) = \frac{g_{NCX} \cdot \left(e^{eta \cdot FVRT} \cdot Na_i^3 \cdot Ca_o - e^{(eta-1) \cdot FVRT} \cdot Na_o^3 \cdot Ca_i\right)}{\left(Na_o^3 + K_{mNa}^3\right) \cdot (Ca_o + K_{mCa}) \cdot \left(1 + k_{sat} \cdot e^{(eta-1) \cdot FVRT}\right)}$$

2.3.6 Sarcolemmal Ca^{2+} pump

$$I_{pCa}(Hinch) = \frac{g_{pCa} \cdot Ca_i}{K_{mpCa} + Ca_i}$$

$$FVRT = \frac{F \cdot V}{R \cdot T}$$

$$FVRT_{Ca} = 2 \cdot FVRT$$

2.3.7 Ca^{2+} independent transient outward K^{+} current r gate

$$r_\infty = \frac{1}{1 + e^{\frac{V+10.6}{-11.42}}}$$

$$\tau_r = \frac{1,000}{45.16 \cdot e^{0.035,77 \cdot (V+50)} + 98.9 \cdot e^{-0.1 \cdot (V+38)}}$$

$$\frac{\mathrm{d}r}{\mathrm{d}time} = \frac{r_\infty - r}{\tau_r}$$

2.3.8 Ca^{2+} independent transient outward K^{+} current s gate

$$s_\infty = \frac{1}{1+e^{\frac{V+45.3}{6.884,1}}}$$

$$\tau_s = 550 \cdot e^{-\left(\frac{V+70}{25}\right)^2} + 49$$

$$\frac{\mathrm{d}s}{\mathrm{d}time} = \frac{s_\infty - s}{\tau_s}$$

2.3.9 Ca^{2+} independent transient outward K^{+} current s slow gate

$$s_{slow_\infty} = \frac{1}{1 + e^{\frac{V+45.3}{6.884,1}}}$$

$$\tau_{s_{slow}} = 3,300 \cdot e^{-\left(\frac{V+70}{30}\right)^2} + 49$$

$$\frac{\mathrm{d}s_{slow}}{\mathrm{d}time} = \frac{s_{slow_\infty} - s_{slow}}{\tau_{s_{slow}}}$$

2.3.10 Ca^{2+} independent transient outward K^{+} current

$$g_t = 0.464,7 \cdot g_t$$

$$E_K = \frac{R \cdot T}{F} \cdot \ln \frac{K_o}{K_i}$$

$$i_t = g_t \cdot r \cdot (a_{Kt} \cdot s + b_{Kt} \cdot s_{slow}) \cdot (V - E_K)$$

2.3.11 Background currents

$$i_{B_{Na}} = g_{B_{Na}} \cdot (V - E_{Na})$$

$$i_{B_K} = g_{B_K} \cdot (V - E_K)$$

2.3.12 Hyperpolarisation activated current y gate

$$y_\infty = \frac{1}{1 + e^{\frac{V+138.6}{10.48}}}$$

$$\tau_y = \frac{1000}{0.11885 \cdot e^{\frac{V+80}{28.37}} + 0.5623 \cdot e^{\frac{V+80}{-14.19}}}$$

$$\frac{\mathrm{d}y}{\mathrm{d}time} = \frac{y_\infty - y}{\tau_y}$$

2.3.13 Hyperpolarisation activated current

$$f_K = 1 - f_{Na}$$

$$i_{f_{Na}} = g_f \cdot y \cdot f_{Na} \cdot (V - E_{Na})$$

$$i_{f_K} = g_f \cdot y \cdot f_K \cdot (V - E_K)$$

$$i_f = i_{f_{Na}} + i_{f_K}$$

2.3.14 Inward rectifier

$$i_{K1} = \left(\frac{48}{e^{\frac{V+37}{25}} + e^{\frac{V+37}{-25}}} + 0.01 \right) \cdot \left(\frac{0.001}{1 + e^{\frac{V-(E_K+76.77)}{-17}}} \right) +$$

$$\frac{g_{K1} \cdot (V - (E_K + 1.73))}{\left(1 + e^{\frac{1.613 \cdot F \cdot (V-(E_K+1.73))}{R \cdot T}}\right) \cdot \left(1 + e^{\frac{K_o - 0.9988}{-0.124}}\right)}$$

2.3.15 $\boldsymbol{Na^+}$ current $\boldsymbol{h}$ gate

$$h_\infty = \frac{1}{1 + e^{\frac{V+76.1}{6.07}}}$$

$$\tau_h = \begin{cases} 0.4537 \cdot \left(1 + e^{\frac{-(V+10.66)}{11.1}}\right) & \text{if } V \geq -40 \\ \dfrac{3.49}{0.135 \cdot e^{\frac{-(V+80)}{6.8}} + 3.56 \cdot e^{0.079 \cdot V} + 310,000 \cdot e^{0.35 \cdot V}} & \text{otherwise} \end{cases}$$

$$\frac{\mathrm{d}h}{\mathrm{d}time} = \frac{h_\infty - h}{\tau_h}$$

2.3.16 $\boldsymbol{Na^+}$ current $\boldsymbol{j}$ gate

$$j_\infty = \frac{1}{1 + e^{\frac{V+76.1}{6.07}}}$$

$$\tau_j =$$

$$\begin{cases} \dfrac{11.63 \cdot \left(1 + e^{-0.1 \cdot (V+32)}\right)}{e^{-0.000,000,253,5 \cdot V}} \text{ if } V \geq -40 \\ \dfrac{3.49}{\dfrac{V+37.78}{1+e^{0.311 \cdot (V+79.23)}} \cdot \left(-127140 \cdot e^{0.2444 \cdot V} - 3.474 \cdot 10^{-5} \cdot e^{-0.04391 \cdot V}\right) + \dfrac{0.1212 \cdot e^{-0.01052 \cdot V}}{1+e^{-0.1378 \cdot (V+40.14)}}} & \text{otherwise} \end{cases}$$

$$\frac{\mathrm{d}j}{\mathrm{d}time} = \frac{j_\infty - j}{\tau_j}$$

2.3.17 Na^+ current m gate

$$m_\infty = \frac{1}{1 + e^{\frac{V+45}{-6.5}}}$$

$$\tau_m = \frac{1.36}{\frac{0.32 \cdot (V+47.13)}{1 - e^{-0.1 \cdot (V+47.13)}} + 0.08 \cdot e^{\frac{-V}{11}}}$$

$$\frac{\mathrm{d}m}{\mathrm{d}time} = \frac{m_\infty - m}{\tau_m}$$

2.3.18 Na^+ current

$$g_{Na} = 1.33 \cdot g_{Na}$$

$$E_{Na} = \frac{R \cdot T}{F} \cdot \ln \frac{Na_o}{Na_i}$$

$$i_{Na} = g_{Na} \cdot m^3 \cdot h \cdot j \cdot (V - E_{Na})$$

2.3.19 Na^+–K^+ pump

$$sigma = \frac{e^{\frac{Na_o}{67.3}} - 1}{7}$$

$$i_{NaK} = i_{NaK_{max}} \cdot \left(\frac{1}{1+0.1245 \cdot e^{\frac{-0.1 \cdot V \cdot F}{R \cdot T}} + 0.0365 \cdot sigma \cdot e^{\frac{-V \cdot F}{R \cdot T}}}\right) \cdot \left(\frac{K_o}{K_o + K_{m_K}}\right) \cdot \left(\frac{1}{1 + \left(\frac{K_{m_{Na}}}{Na_i}\right)^4}\right)$$

2.3.20 Steady state outward K^+ current r_{ss} gate

$$r_{ss_\infty} = \frac{1}{1 + e^{\frac{V+11.5}{-11.82}}}$$

$$\tau_{r_{ss}} = \frac{10000}{45.16 \cdot e^{0.035,77 \cdot (V+50)} + 98.9 \cdot e^{-0.1 \cdot (V+38)}}$$

$$\frac{\mathrm{d}r_{ss}}{\mathrm{d}time} = \frac{r_{ss_\infty} - r_{ss}}{\tau_{r_{ss}}}$$

2.3.21 Steady state outward K^+ current s_{ss} gate

$$s_{ss_\infty} = \frac{1}{1 + e^{\frac{V+87.5}{10.3}}}$$

$$\tau_{s_{ss}} = 2100$$

$$\frac{\mathrm{d}s_{ss}}{\mathrm{d}time} = \frac{s_{ss_\infty} - s_{ss}}{\tau_{s_{ss}}}$$

2.3.22 Steady state outward K^+ current

$$i_{ss} = g_{ss} \cdot r_{ss} \cdot s_{ss} \cdot (V - E_K)$$

2.4 Ca^{2+} DYNAMICS

2.4.1 Ca^{2+} release unit (CaRU) transitions

$$expVL = e^{\frac{V-V_L}{del_{VL}}}$$

$$t_R = 1.17 \cdot t_L$$

$$\alpha_p = \frac{expVL}{t_L \cdot (expVL + 1)}$$

$$\alpha_m = \frac{\phi_L}{t_L}$$

$$\beta_{poc} = \frac{C_{oc}^2}{t_R \cdot \left(C_{oc}^2 + K_{RyR}^2\right)}$$

$$\beta_{pcc} = \frac{Ca_i^2}{t_R \cdot \left(Ca_i^2 + K_{RyR}^2\right)}$$

$$\beta_m = \frac{\phi_R}{t_R}$$

$$\epsilon_{pco} = \frac{C_{co} \cdot (expVL + a)}{\tau_L \cdot K_L \cdot (expVL + 1)}$$

$$\epsilon_{pcc} = \frac{Ca_i \cdot (expVL + a)}{\tau_L \cdot K_L \cdot (expVL + 1)}$$

$$\epsilon_m = \frac{b \cdot (expVL + a)}{\tau_L \cdot (b \cdot expVL + a)}$$

$$\mu_{poc} = \frac{C_{oc}^2 + c \cdot K_{RyR}^2}{\tau_R \cdot \left(C_{oc}^2 + K_{RyR}^2\right)}$$

$$\mu_{pcc} = \frac{Ca_i^2 + c \cdot K_{RyR}^2}{\tau_R \cdot \left(Ca_i^2 + K_{RyR}^2\right)}$$

$$\mu_{moc} = \frac{\theta_R \cdot d \cdot \left(C_{oc}^2 + c \cdot K_{RyR}^2\right)}{\tau_R \cdot \left(d \cdot C_{oc}^2 + c \cdot K_{RyR}^2\right)}$$

$$\mu_{mcc} = \frac{\theta_R \cdot d \cdot \left(Ca_i^2 + c \cdot K_{RyR}^2\right)}{\tau_R \cdot \left(d \cdot Ca_i^2 + c \cdot K_{RyR}^2\right)}$$

2.4.2 CaRU reduced states

$$r_1 = y_{oc} \cdot \mu_{poc} + y_{cc} \cdot \mu_{pcc}$$

$$r_2 = \frac{\alpha_p \cdot \mu_{moc} + \alpha_m \cdot \mu_{mcc}}{\alpha_p + \alpha_m}$$

$$r_3 = \frac{\beta_m \cdot \mu_{pcc}}{\beta_m + \beta_{pcc}}$$

$$r_4 = \mu_{mcc}$$

$$r_5 = y_{co} \cdot \epsilon_{pco} + y_{cc} \cdot \epsilon_{pcc}$$

$$r_6 = \epsilon_m$$

$$r_7 = \frac{\alpha_m \cdot \epsilon_{pcc}}{\alpha_p + \alpha_m}$$

$$r_8 = \epsilon_m$$

$$z_4 = 1 - z_1 - z_2 - z_3$$

$$\frac{\mathrm{d}z_1}{\mathrm{d}time} = -\left(r_1 + r_5\right) \cdot z_1 + r_2 \cdot z_2 + r_6 \cdot z_3$$

$$\frac{\mathrm{d}z_2}{\mathrm{d}time} = r_1 \cdot z_1 - \left(r_2 + r_7\right) \cdot z_2 + r_8 \cdot z_4$$

$$\frac{\mathrm{d}z_3}{\mathrm{d}time} = r_5 \cdot z_1 - \left(r_6 + r_3\right) \cdot z_3 + r_4 \cdot z_4$$

2.4.3 CaRU states

$$denom = (\alpha_p + \alpha_m) \cdot ((\alpha_m + \beta_m + \beta_{poc}) \cdot (\beta_m + \beta_{pcc}) + \alpha_p \cdot (\beta_m + \beta_{poc}))$$

$$y_{oc} = \frac{\alpha_p \cdot \beta_m \cdot (\alpha_p + \alpha_m + \beta_m + \beta_{pcc})}{denom}$$

$$y_{co} = \frac{\alpha_m \cdot (\beta_{pcc} \cdot (\alpha_m + \beta_m + \beta_{poc}) + \beta_{poc} \cdot \alpha_p)}{denom}$$

$$y_{oo} = \frac{\alpha_p \cdot (\beta_{poc} \cdot (\alpha_p + \beta_m + \beta_{pcc}) + \beta_{pcc} \cdot \alpha_m)}{denom}$$

$$y_{cc} = \frac{\alpha_m \cdot \beta_m \cdot (\alpha_m + \alpha_p + \beta_m + \beta_{poc})}{denom}$$

$$y_{ci} = \frac{\alpha_m}{\alpha_p + \alpha_m}$$

$$y_{oi} = \frac{\alpha_p}{\alpha_p + \alpha_m}$$

$$y_{ic} = \frac{\beta_m}{\beta_{pcc} + \beta_m}$$

$$y_{io} = \frac{\beta_{pcc}}{\beta_{pcc} + \beta_m}$$

$$y_{ii} = 1 - y_{oc} - y_{co} - y_{oo} - y_{cc} - y_{ci} - y_{ic} - y_{oi} - y_{io}$$

2.4.4 DS Calcium Concentrations

$$C_{cc} = Ca_i$$

$$C_{co} = \frac{Ca_i + \frac{J_R}{g_D} \cdot Ca_{SR}}{1 + \frac{J_R}{g_D}}$$

$$C_{oc} = \begin{cases} \frac{Ca_i + \frac{\frac{J_L}{g_D} \cdot Ca_o \cdot FVRT_{Ca} \cdot e^{-FVRT_{Ca}}}{1 - e^{-FVRT_{Ca}}}}{1 + \frac{\frac{J_L}{g_D} \cdot FVRT_{Ca}}{1 - e^{-FVRT_{Ca}}}} & \text{if } |FVRT_{Ca}| > 10^{-9} \\ \frac{Ca_i + \frac{J_L}{g_D} \cdot Ca_o}{1 + \frac{J_L}{g_D}} & \text{otherwise} \end{cases}$$

$$C_{oo} = \begin{cases} \frac{Ca_i + \frac{J_R}{g_D} \cdot Ca_{SR} + \frac{\frac{J_L}{g_D} \cdot Ca_o \cdot FVRT_{Ca} \cdot e^{-FVRT_{Ca}}}{1 - e^{-FVRT_{Ca}}}}{1 + \frac{J_R}{g_D} + \frac{\frac{J_L}{g_D} \cdot FVRT_{Ca}}{1 - e^{-FVRT_{Ca}}}} & \text{if } |FVRT_{Ca}| > 10^{-9} \\ \frac{Ca_i + \frac{J_R}{g_D} \cdot Ca_{SR} + \frac{J_L}{g_D} \cdot Ca_o}{1 + \frac{J_R}{g_D} + \frac{J_L}{g_D}} & \text{otherwise} \end{cases}$$

2.4.5 LCC and RyR fluxes

$$J_{Rco} = \frac{J_R \cdot (Ca_{SR} - Ca_i)}{1 + \frac{J_R}{g_D}}$$

$$J_{Roo} =$$

$$\begin{cases} \dfrac{J_R \cdot \left(Ca_{SR} - Ca_i + \dfrac{\dfrac{J_L}{g_D} \cdot FVRT_{Ca}}{1 - e^{-FVRT_{Ca}}} \cdot \left(Ca_{SR} - Ca_o \cdot e^{-FVRT_{Ca}} \right) \right)}{1 + \dfrac{J_R}{g_D} + \dfrac{\dfrac{J_L}{g_D} \cdot FVRT_{Ca}}{1 - e^{-FVRT_{Ca}}}} & \text{if } |FVRT_{Ca}| > 10^{-5} \\ \dfrac{J_R \cdot \left(Ca_{SR} - Ca_i + \dfrac{\dfrac{J_L}{g_D} \cdot 0.00001}{1 - e^{-0.000{,}01}} \cdot \left(Ca_{SR} - Ca_o \cdot e^{-0.000{,}01} \right) \right)}{1 + \dfrac{J_R}{g_D} + \dfrac{\dfrac{J_L}{g_D} \cdot 0.00001}{1 - e^{-0.000{,}01}}} & \text{otherwise} \end{cases}$$

$$J_{Loc} = \begin{cases} \dfrac{\dfrac{J_L \cdot FVRT_{Ca}}{1 - e^{-FVRT_{Ca}}} \cdot \left(Ca_o \cdot e^{-FVRT_{Ca}} - Ca_i \right)}{1 + \dfrac{\dfrac{J_L}{g_D} \cdot FVRT_{Ca}}{1 - e^{-FVRT_{Ca}}}} & \text{if } |FVRT_{Ca}| > 10^{-5} \\ \dfrac{\dfrac{J_L \cdot 0.00001}{1 - e^{-0.00001}} \cdot \left(Ca_o \cdot e^{-0.000{,}01} - Ca_i \right)}{1 + \dfrac{\dfrac{J_L}{g_D} \cdot 0.00001}{1 - e^{-0.00001}}} & \text{otherwise} \end{cases}$$

$$J_{Loo} =$$

$$\begin{cases} \dfrac{\dfrac{J_L \cdot FVRT_{Ca}}{1 - e^{-FVRT_{Ca}}} \cdot \left(Ca_o \cdot e^{-FVRT_{Ca}} - Ca_i + \dfrac{J_R}{g_D} \cdot \left(Ca_o \cdot e^{-FVRT_{Ca}} - Ca_{SR} \right) \right)}{1 + \dfrac{J_R}{g_D} + \dfrac{\dfrac{J_L}{g_D} \cdot FVRT_{Ca}}{1 - e^{FVRT_{Ca}}}} & \text{if } |FVRT_{Ca}| > 10^{-5} \\ \dfrac{\dfrac{J_L \cdot 0.00001}{1 - e^{-0.00001}} \cdot \left(Ca_o \cdot e^{-0.00001} - Ca_i + \dfrac{J_R}{g_D} \cdot \left(Ca_o \cdot e^{-0.00001} - Ca_{SR} \right) \right)}{1 + \dfrac{J_R}{g_D} + \dfrac{\dfrac{J_L}{g_D} \cdot 0.000,01}{1 - e^{-0.00001}}} & \text{otherwise} \end{cases}$$

2.4.6 LCC current

$$J_{L1} = J_{Loo} \cdot y_{oo} + J_{Loc} \cdot y_{oc}$$

$$J_{L2} = \frac{J_{Loc} \cdot \alpha_p}{\alpha_p + \alpha_m}$$

$$I_{LCC}(Hinch) = \frac{(z_1 \cdot J_{L1} + z_2 \cdot J_{L2}) \cdot N_{RU}}{V_{myo}}$$

2.4.7 RyR current

$$J_{R1} = y_{oo} \cdot J_{Roo} + J_{Rco} \cdot y_{co}$$

$$J_{R3} = \frac{J_{Rco} \cdot \beta_{pcc}}{\beta_m + \beta_{pcc}}$$

$$I_{RyR}(Hinch) = \frac{(z_1 \cdot J_{R1} + z_3 \cdot J_{R3}) \cdot N_{RU}}{V_{myo}}$$

2.4.8 Ca^{2+} pumping from the cytoplasm to the SR

$$I_{SERCA} = \frac{g_{SERCA} \cdot Ca_i^2}{K_{SERCA}^2 + Ca_i^2}$$

2.4.9 SR Ca^{2+} leak current

$$I_{SR} = g_{SRl} \cdot (Ca_{SR} - Ca_i)$$

2.4.10 Calmodulin Ca^{2+} buffer

$$\beta_{CMDN} = \left(1 + \frac{k_{CMDN} \cdot B_{CMDN}}{(k_{CMDN} + Ca_i)^2}\right)^{-1}$$

2.4.11 Average fraction of the attached cross-bridges per one $CaTnC$ complex

$$N_A = \frac{TnC_{tot} \cdot N \cdot f_A}{L_{oz} \cdot CaTnC}$$

2.4.12 Dependence defining cooperativity of the contractile proteins

$$\Pi_{N_A} = \begin{cases} 1 & \text{if } N_A \leq 0 \\ \Pi_{min}^{N_A} & \text{if } N_A \leq 1 \\ \Pi_{min} & \text{otherwise} \end{cases}$$

2.4.13 $CaTnC$, Ca^{2+}-troponin C complexes complexes

$$\frac{\mathrm{d}CaTnC}{\mathrm{d}time} = a_{on} \cdot (TnC_{tot} - CaTnC) \cdot Ca_i - a_{off} \cdot e^{-k_A \cdot CaTnC} \cdot \Pi_{N_A} \cdot CaTnC$$

2.5 Mechanical block of the model

Forces

2.5.1 Contractile element (sarcomere) (CE) force

$$F_{CE} = \lambda \cdot p_v \cdot N$$

2.5.2 Series elastic element (SE) force

$$F_{SE} = \beta_1 \cdot \left(e^{\alpha_1 \cdot (l_2 - l_1)} - 1\right)$$

2.5.3 Parallel elastic element (PE) force

$$F_{PE} = \alpha_2 \cdot l_2 + \beta_2$$

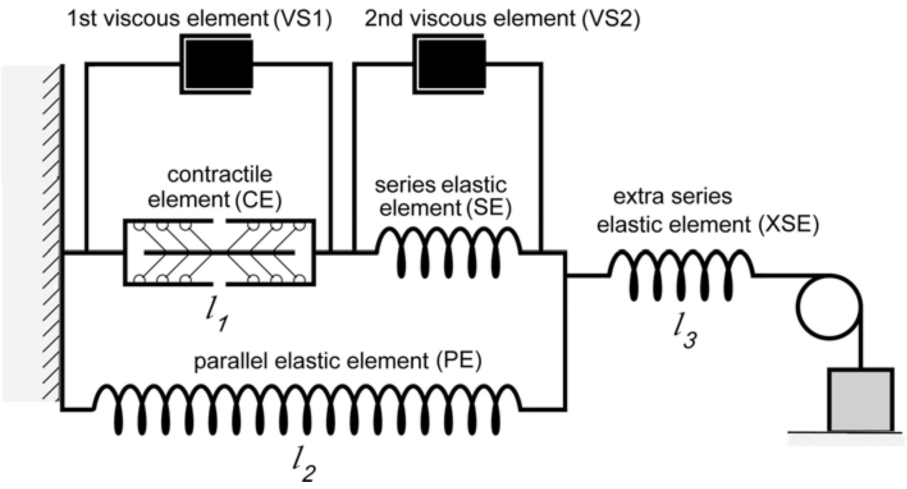

Figure S2. Rheological scheme of the model

2.5.4 Extra series elastic element ($\boldsymbol{XSE}$) force

$$F_{XSE} = \alpha_3 \cdot l_3 + \beta_3$$

2.5.5 Viscous elements ($\boldsymbol{VS1}$, $\boldsymbol{VS2}$) forces

$$F_{VS_1} = k_{P_{vis}} \cdot v$$

$$k_{P_{vis}} = \begin{cases} \beta_{vp_l} \cdot e^{\alpha_{vp_l} \cdot l_1} & \text{if } v \leq 0 \\ \beta_{vp_s} \cdot e^{\alpha_{vp_s} \cdot l_1} & \text{otherwise} \end{cases}$$

$$F_{VS_2} = k_{S_{vis}} \cdot (w - v)$$

$$k_{S_{vis}} = \begin{cases} \beta_{vs_l} \cdot e^{\alpha_{vs_l} \cdot (l_2 - l_1)} & \text{if } w \leq v \\ \beta_{vs_s} \cdot e^{\alpha_{vs_s} \cdot (l_2 - l_1)} & \text{otherwise} \end{cases}$$

2.5.6 Force developed by the cardiomyocyte

$$F_{sample} = F_{XSE}$$

2.5.7 Length

$$l = l_2 + l_3$$

$$\frac{\mathrm{d}l_1}{\mathrm{d}time} = v$$

$$\frac{\mathrm{d}l_2}{\mathrm{d}time} = w$$

The variable v is solution of the algebraic equation:

$$F_{CE} + F_{VS1} = F_{SE} + F_{VS2}$$

The variable l_2 is solution of the algebraic equation:

$$F_{SE} + F_{VS2} + F_{PE} = F_{XSE}$$

2.5.8 Average crossbridge force

$$v_1 = x_1 \cdot v_{max}$$

$$\gamma = \frac{a_p \cdot d_h \cdot \left(\frac{v_1}{v_{max}}\right)^2}{3 \cdot a_p \cdot d_h - \frac{(a_p + 1) \cdot v_1}{v_{max}}}$$

$$P_{star} = \begin{cases} \frac{a_p \cdot \left(1 + \frac{v}{v_{max}}\right)}{a_p - \frac{v}{v_{max}}} & \text{if } v \leq 0 \\ 1 + d_h - \frac{d_h^2 \cdot a_p}{\frac{a_p \cdot d_h}{\gamma} \cdot \left(\frac{v}{v_{max}}\right)^2 + \frac{(a_p + 1) \cdot v}{v_{max}} + a_p \cdot d_h} & \text{otherwise} \end{cases}$$

$$G_{star} = \begin{cases} 1 + \frac{0.6 \cdot v}{v_{max}} & \text{if } (v \leq 0) \\ \frac{P_{star}}{\frac{(0.4 \cdot a_p + 1) \cdot v}{a_p \cdot v_{max}} + 1} & \text{if } (0 < v) \text{ and } (v \leq v_1) \\ \frac{P_{star} \cdot e^{-\alpha_G \cdot \left(\frac{v - v_1}{v_{max}}\right)^{\alpha_P}}}{\frac{(0.4 \cdot a_p + 1) \cdot v}{a_p \cdot v_{max}} + 1} & \text{otherwise} \end{cases}$$

$$p_v = \frac{P_{star}}{G_{star}}$$

2.5.9 Crossbridge kinetics

$$M_A = \frac{\left(\frac{CaTnC}{TnC_{tot}}\right)^{\mu} \cdot \left(1 + k_{\mu}^{\mu}\right)}{\left(\frac{CaTnC}{TnC_{tot}}\right)^{\mu} + k_{\mu}^{\mu}}$$

$$temp_{n1} = (g_1 \cdot l_1 + g_2) \cdot \left(n1_A + \frac{n1_K - n1_A}{\left(n1_C + n1_Q \cdot e^{-n1_B \cdot l_1}\right)^{\frac{1}{n1_\nu}}}\right)$$

$$n_1 = \begin{cases} 0 & \text{if } temp_{n1} < 0 \\ temp_{n1} & \text{if } temp_{n1} < 1 \\ 1 & \text{otherwise} \end{cases}$$

$$L_{oz} = \begin{cases} \dfrac{l_1 + S_0}{S_{046} + S_0} & \text{if } l_1 \leq S_{055} \\ \\ \dfrac{S_0 + S_{055}}{S_{046} + S_0} & \text{otherwise} \end{cases}$$

$$v_{st} = x_{st} \cdot v_{max}$$

$$q_v = \begin{cases} q_1 - \dfrac{q_2 \cdot v}{v_{max}} & \text{if } v \leq 0 \\ \\ \dfrac{(q_4 - q_3) \cdot v}{v_{st}} + q_3 & \text{if } (v \leq v_{st}) \text{ and } (0 < v) \\ \\ \dfrac{q_4}{\left(1 + \dfrac{\beta_Q \cdot (v - v_{st})}{v_{max}}\right)^{\alpha_Q}} & \text{otherwise} \end{cases}$$

$$k_{p_v} = \kappa \cdot \kappa_0 \cdot q_v \cdot m_0 \cdot G_{star}$$

$$k_{m_v} = \kappa_0 \cdot q_v \cdot (1 - \kappa \cdot m_0 \cdot G_{star})$$

$$\frac{\mathrm{d}N}{\mathrm{d}time} = k_{p_v} \cdot M_A \cdot n_1 \cdot L_{oz} \cdot (1 - N) - k_{m_v} \cdot N$$

REFERENCES

Pandit SV, Clark RB, Giles WR, Demir SS. A mathematical model of action potential heterogeneity in adult rat left ventricular myocytes. *Biophysical journal* **81** (2001) 3029–3051.

Hinch R, Greenstein J, Tanskanen A, Xu L, Winslow R. A simplified local control model of calcium-induced calcium release in cardiac ventricular myocytes. *Biophysical journal* **87** (2004) 3723–3736.

Sulman T, Katsnelson LB, Solovyova O, Markhasin VS. Mathematical modeling of mechanically modulated rhythm disturbances in homogeneous and heterogeneous myocardium with attenuated activity of na+–k+ pump. *Bulletin of mathematical biology* **70** (2008) 910–949.

Terkildsen JR, Niederer S, Crampin EJ, Hunter P, Smith NP. Using physiome standards to couple cellular functions for rat cardiac excitation–contraction. *Experimental physiology* **93** (2008) 919–929.

Partial conversion from CellML 1.0 to TEX was done using COR (0.9.31.1409)
Copyright 2002-2018 Dr Alan Garny
